# Transient crystallisation of rare earth carbonates during the hydrothermal oxidation of siderite†

**DOI:** 10.1039/d4ra05212a

**Published:** 2024-11-05

**Authors:** Maddin M., Rateau R., Szucs A. M., Terribili L., Rodriguez-Blanco J. D.

**Affiliations:** a Department of Geology, School of Natural Sciences, Trinity College Dublin Dublin 2 Ireland maddinm@tcd.ie; b iCRAG, Department of Geology, School of Natural Sciences, Trinity College Dublin Dublin 2 Ireland

## Abstract

The researchers investigated the interaction between multi-component rare earth element-bearing aqueous solutions and siderite grains under hydrothermal conditions. Our study investigates the interaction between multi-component rare earth element (REE; La, Ce, Pr, Nd, Dy)-bearing aqueous solutions and siderite (FeCO_3_) grains under hydrothermal conditions (50–205 °C). The results revealed a solution-mediated mineral replacement reaction that occurs *via* a multi-step crystallisation pathway involving the formation of iron oxides (goethite, α-FeO(OH), and hematite, Fe_2_O_3_), metastable REE-bearing minerals (kozoite, REE(CO_3_)(OH), and bastnasite, REE(CO_3_)(OH,F)), and cerianite (CeO_2_). Siderite stability, dissolution, and subsequent mineral formation are temperature and pH-dependent. At low temperatures, REE carbonate formation is inhibited by a goethite coating, creating a partial equilibrium situation. Higher temperatures increase dissolution rates and enable kozoite and bastnasite formation. The redox behaviour of Fe and Ce combined with the temperature, and the availability of CO_3_^2−^ govern this crystallisation sequence. Continued oxidation promotes decarbonation processes by acidifying the aqueous solution, dissolving all carbonates, and resulting in hematite and cerianite crystallisation as thermodynamically stable phases. Understanding iron carbonate, oxide and REE interactions can inform new resource targets and improve recovery and separation techniques.

## Abstract 2

The researchers investigated the interaction between multi-component rare earth element-bearing aqueous solutions and siderite grains under hydrothermal conditions.

## Graphical abstract

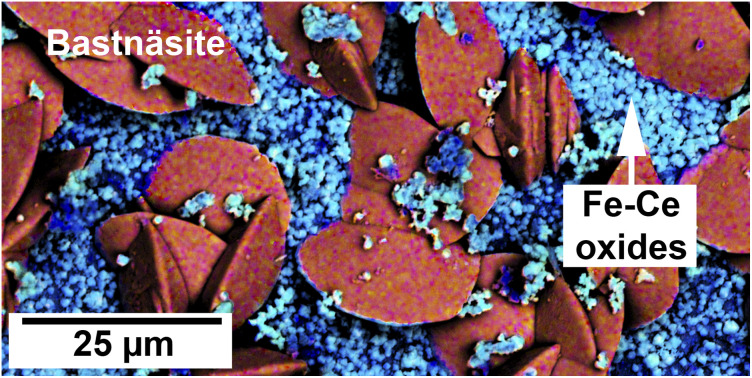

## Introduction

Due to their application in a range of green technologies and renewable energy, the demand for rare earth elements (REE) is ever increasing.^1,2^ While the development of recycling REE from end-products has progressed, we are still heavily reliant on natural, mineable sources.^3^ Currently, China is host to one-third of all known REE deposits and processes 97% of the world's REE + Y.^3,4^

Presently, commercially significant REE deposits are associated with magmatic processes related to alkaline igneous rocks and carbonatites.^5^ As of 2016, there were approximately 50 advanced REE exploration projects active outside of China, 20 of which are targeting carbonate igneous rocks (carbonatites).^6^ Carbonatites and alkaline–carbonatite complexes have been identified as significant sources of REE and Nb, as well as hosting substantial deposits of apatite, vermiculite, copper, fluorite, thorium, uranium, and iron.^7^ The genesis of carbonatite-associated REE deposits, a vital global REE source, is therefore a subject of strong interest and investigation.^8^

In general, carbonatites are igneous rocks derived from the mantle and have a carbonate mineral volume of >50% and SiO_2_ <20 wt%.^9^ Their formation occurs through the immiscibility of carbonate–silicate magma and through the fractional crystallisation of carbonate minerals from carbonatite magma. The main source of these ore-forming elements comes from primitive mantle with an additional contribution from REE carrying crustal materials.^5^ The REE form complexes and migrate through the magmatic-hydrothermal system. Changes in temperature, pressure, pH and fluid composition allow their precipitation and mineralization. The formation of the world's largest REE deposit, Bayan Obo REE-Nb-Fe in China, has been linked to prolonged fluxing of carbonatite by subduction-derived fluids, and highlights the intricate interplay of geological processes involved in REE enrichment.^10^ It is characterized by a light rare earth (LREE) enrichment and is also hosted in hematite-bearing iron ores with high REE concentrations.^11^

Investigations into the magmatic-hydrothermal processes in carbonatite-related REE deposits have offered insights into the enrichment mechanisms of REE, such as the Saint-Honoré carbonatite complex in Canada^12^ and the Weishan carbonatite-related REE deposit in China.^13^ For example, in Weishan, two enrichment processes were responsible for the formation of the deposit: (i) the production of mineralized carbonatite and (ii) the subsequent enrichment by magmatic-hydrothermal processes. Fractures in the deposit promoted the circulation of ore-forming fluids and provided the space necessary for REE precipitation. Concomitantly, high F^−^ and SO_4_^2−^ concentrations in the ore-forming fluids are thought to have facilitated REE transport and deposition *via* the formation of stable complexes.^13^

The behaviour of fluids under high-pressure conditions can influence the phase transitions of carbonatitic melts and the subsequent formation of hydrothermal brines. It has been demonstrated that under high-pressure and high-temperature conditions, the transition from carbonatitic melts to hydrothermal brines occurs, which can lead to the concentration of REE in these brines.^14^ The pressure conditions can also affect the stability of mineral phases, influencing which REE-bearing minerals are formed during the cooling and crystallization processes.

The pH of the fluid environment is another crucial factor influencing REE mineralization. Acidic conditions can enhance the solubility of REE and promote their mobility, while neutral to alkaline conditions may stabilize certain REE-bearing minerals. Anenburg and Mavrogenes highlighted that the transport of REE influenced by the pH of the fluids, with different REE complexes forming under varying pH conditions.^15^ Furthermore, the presence of ligands such as F^−^, Cl^−^, and CO_3_^2−^ can significantly affect the solubility and transport of REE, with acidic conditions often favouring the dissolution of REE minerals and their subsequent transport in solution.

If REE carbonates are heated at temperatures up to their melting points, several processes would occur, dependent on the specific REE and temperature. Initially, dehydration would happen if the carbonates contained structural water or –OH groups, with heavier REE requiring temperatures above 700 °C for water release. As temperatures rise, REE carbonates would undergo phase transitions, transforming into anhydrous oxycarbonates and eventually into oxides.^16,17^ This decarbonation process, releasing CO_2_, occurs at around 450–550 °C for oxycarbonates, with heavier REE requiring higher temperatures due to their smaller ionic radii and ability to retain some minor amounts of structural water.^16^ Formation of solid solutions is expected, but differences in ionic sizes would lead to distinct hexagonal, monoclinic, or cubic structures.^16,18^ At more elevated temperatures, the REE oxides would melt, and the behaviour of REE in the melt would depend on their partitioning, melt composition, and interactions with other elements from the rock.

The composition of the fluids involved in carbonatite formation is critical for the concentration of REE. Fluids rich in specific ligands (*e.g.*, F^−^, Cl^−^, SO_4_^2−^) can enhance the mobility of REE and facilitate their precipitation as economically viable minerals. Pokrovsky *et al.*, 2002 ^19^ noted that the fluids involved in REE transport and deposition are characterized by high activities of ligands and brines, which play a significant role in the mineralization processes. Additionally, the presence of carbonate-rich fluids can lead to the formation of REE-bearing minerals through complexation and precipitation reactions.^5^ The interaction of these fluids with the host rocks can also lead to metasomatic processes that enrich the REE content in carbonatites.^20^

There are also several examples of REE-rich carbonatite complexes containing high REE concentrations connected to hematite-bearing rocks and iron ores,^11^ one being the Mountain Pass complex in California. Another example is the Fen complex, Telemark, southeast Norway where REE concentrations of up to 15 000 ppm have been recorded and originate from hematite–carbonatite and iron ores.^21–23^ The Fen complex was formed by the alteration of ankerite ferrocarbonatite which included the oxidation of ferrous minerals in the primary ferrocarbonatite assemblages.^21,24–26^ One of the largest concentrations of Fe-oxides on Earth is the Olympic Dam Cu–U–Au–Ag iron oxide copper–gold (IOCG-type) deposit, in South Australia,^27^ with hematite being the most abundant mineral in the deposit.^26^ REE-rich hematite carbonatites are found in several carbonatite complexes worldwide and are commonly associated with ankerite [Ca(Fe, Mg, Mn)(CO_3_)_2_]- and siderite (FeCO_3_)-bearing carbonatites.^11^

In this experimental study we aim to better understand the formation of rare earth carbonates during the oxidation and replacement of siderite. Of particular interest is the crystallization of REE carbonates in the presence of multiple foreign ions during the oxidation process as well as the extent to which REE are incorporated into the iron oxides that may form. Therefore, we have mimicked natural systems by carrying out replacement experiments using multi-component REE aqueous solutions and iron carbonate host grains of siderite, which are commonly associated with carbonatite ore deposits.

## Methods

The interaction of siderite (FeCO_3_) grains with multi-component REE (La, Ce, Pr, Nd and Dy) aqueous solutions was investigated at hydrothermal conditions (50–205 °C). Siderite crystals were crushed in a ceramic mortar and sieved to extract 0.5–1.0 mm clasts. Five single REE bearing solutions were prepared, La, Ce, Pr, Nd and Dy, with a total REE concentration of 50 mM. These solutions were produced *via* the dissolution of single REE nitrate salts, REE(NO_3_)_3_·6H_2_O (Sigma-Aldrich, 99.9% purity), in de-ionized water (18.18 MΩ cm). These five specific REE were chosen as they are representative of both light (La, Ce, Pr, and Nd) and heavy (Dy) REE. This suit of REE from La to Dy also represents 72% of the ionic radii of the lanthanides as well as being some of the most abundant REE in the earth's crust.^28^ In order to understand the effect of ionic radii of the five REEs and to what extent these ions are taken up by the siderite host, two different sets of experiments were conducted, one with equal concentrations of the five REE and one with the concentrations of the five REE normalized to the commonly used Post Archean Australian Shale standard (PAAS)^29^ to mimic the REE concentrations found in continental crust and natural geologic fluids. For the equal concentration experiments, 4 mL of each of the five REE were used to give a total solution volume of 20 mL for each experiment. For the PAAS experiments, a 1 L bulk solution was made and 20 mL of this was used for each experiment.

For each experiment, 0.1 g of siderite grains were added to 20 mL of each of the 50 mM REE-bearing solutions and placed in 25 mL Teflon-lined and capped stainless-steel autoclaves (for the 165 and 205 °C experiments). For the 50 °C experiments, narrow mouth Nalgene polypropylene PPCO bottles (Thermo Fisher Scientific) were used. The reactors were then placed in a pre-heated oven at 50, 165, and 205 °C. Solid samples were then extracted using a sterilized metal spatula at increasing time intervals from 24 hours to 12 weeks. The samples were placed in plastic Eppendorf tubes and dried in a 30 °C oven for a minimum of 1 hour. The samples were then inspected several days later before preparing them for XRD and SEM analysis.

In order to identify and quantify the formation of crystalline solids present in our samples, several grains were selected from each experiment ensuring that each time and temperature variable was represented. The selected grains were ground to a consistently fine powder using an agate pestle and mortar. The minerals were identified and quantified with powder X-ray diffraction (XRD). Conventional powder XRD patterns were collected using a Siemens/Bruker D5000 powder X-ray diffractometer (CuKα radiation, 0.01° per step from 5 to 60° 2*θ* at 0.2° min^−1^; 4.5 hours scans per sample). Identification of crystalline phases was carried out with the Diffract Suite EVA software from Bruker in combination with the Powder Data File (PDF-4, the International Centre for Diffraction Data).^30^ Pattern-matching refinement and quantification of crystalline phases were carried out with the Rietveld refinement software TOPAS.^31^ Finally, scanning electron microscopy (SEM) was used to obtain high resolution images to characterize changes in the morphology of the siderite host grains and identify newly formed phases. Analyses were conducted in the iCRAG Lab at Trinity College Dublin using a TIGER S8000 FEG-SEM operating under high vacuum conditions and equipped with two Oxford X-Max 170 mm^2^ EDS detectors and an X4 pulse processor running the Oxford Aztec analysis software.

Two methods of sample preparation were employed: (i) standard SEM sample stubs with intact grains mounted to examine surface morphology, and (ii) polished epoxy resin pucks that provided a cross section of the grains, allowing for the identification of potential differences in spatial distribution of elements and any internal chemical textures. The epoxy mounts (2.5 cm wide) were made using Epoxy resin (Struers Epofix) mixed with the accompanying hardener and then polished using a three-step process (Struers Diapro, 6 and 1 μm) to expose the internal cross section of the grains. The pucks were then cleaned in an ultrasonic bath of deionized water to remove any residual polishing fluid and then dried. Both the stubs and the pucks were then coated with either carbon (for elemental mapping and analysis) or gold (for high-resolution imaging). Analysis at a working distance of 15 mm was performed using an accelerated voltage of 20 kV, while imaging carried out at a working distance of 5 mm was performed using an accelerating voltage of 10 kV. The images and maps were processed using the Aztec v6.1 X-ray microanalysis software suit (Oxford Instruments).

Finally, the saturation indexes of solid phases during the equilibration of the REE-bearing aqueous solutions with respect to siderite were calculated with the hydro-geochemical code PHREEQC^32^ using the LLNL database and the solubility products of REE-bearing carbonates determined by ref. 33 and 34.

## Results

The analysis of all solid samples obtained from the replacement experiments resulted in the formation of surface precipitates that partially or totally replaced the siderite host grain. The combination of powder X-ray diffraction (XRD), scanning electron microscopy (SEM) and energy dispersive X-ray spectroscopy (EDS) allowed us to identify and quantify the newly formed phases and interpret the mechanisms responsible for the alteration and decomposition of the siderite grains and the precipitation of the newly formed phases.

### Powder X-ray diffraction (XRD)

The interaction between siderite and multi-component rare earth element (La, Ce, Pr, Nd and Dy) – bearing aqueous solutions resulted in the formation of iron oxides, goethite (α-FeO(OH); PDF 00-003-0249) and hematite (Fe_2_O_3_; PDF 00-024-0072), REE-bearing minerals kozoite (REE(CO_3_)(OH); PDF 04-017-1451) and hydroxylbastnasite (REE(CO_3_)(OH,F); PDF 00-027-1295) and the rare earth oxide cerianite (CeO_2_; PDF 00-004-0593). The full quantitative XRD data are presented in Table 1. These newly formed phases were observed as surface precipitates that partially or fully covered the siderite host mineral. In some experiments, siderite was fully replaced from the periphery inwards.

**Table 1:** Experimental conditions, identities and morphologies of the solid rare earth carbonate and oxide phases formed during the interaction of siderite with multi-component (La, Ce, Pr, Nd, and Dy) REE-bearing aqueous solutions (equal concentration and PAAS experiments) at 50, 165, and 205 °C^a^

Equal concentration experiments					PAAS experiments			
*T* (°C)	Time (days)	% phase consumed	Phase formed	Morphology	Time (days)	% phase consumed	Phase formed	Morphology
50	1	<1	Goethite	Flakes	1	<1	Goethite	Flakes
	2	<1			2	<1		
	3	<1			3	<1		
	4	<1			4	<1		
	12	<1			12	<1		
	56	<1			56	<1		
	84	<1			84	<1		
165	1	40	33% Hem, 6% Koz, <1% goeth	Koz (spindle-shaped prisms), Hem (pseudohexagonal prisms), Cer (cubes, bipyramidal prisms), HB (triangular prisms)	1	70	46% Hem, 15% HB, 9% goeth	Hem (pseudohexagonal prisms), Cer (cubes, bipyramidal prisms), HB (triangular prisms)
	7	100	87% Hem, 3% HB, 9% Cer, <1% goeth		7	100	73% Hem, 20% Cer, 7% goeth	
	14	100	77% Hem, 2% Cer, 21% goeth		14	100	82% Hem, 18% Cer	
205	1	84	77% Hem, 4% HB, 3% Cer	Hem (pseudohexagonal prisms), Cer (cubes, bipyramidal prisms)	1	100	83% Hem, 17% Cer	Hem (pseudohexagonal prisms), Cer (cubes, bipyramidal prisms)
	7	100	95% Hem, 5% Cer		7	100	94% Hem, 6% Cer	

a Goethite (goeth), hematite (Hem), kozoite (koz), hydroxylbastnasite (HB), cerianite (Cer).

**Fig. 1:**
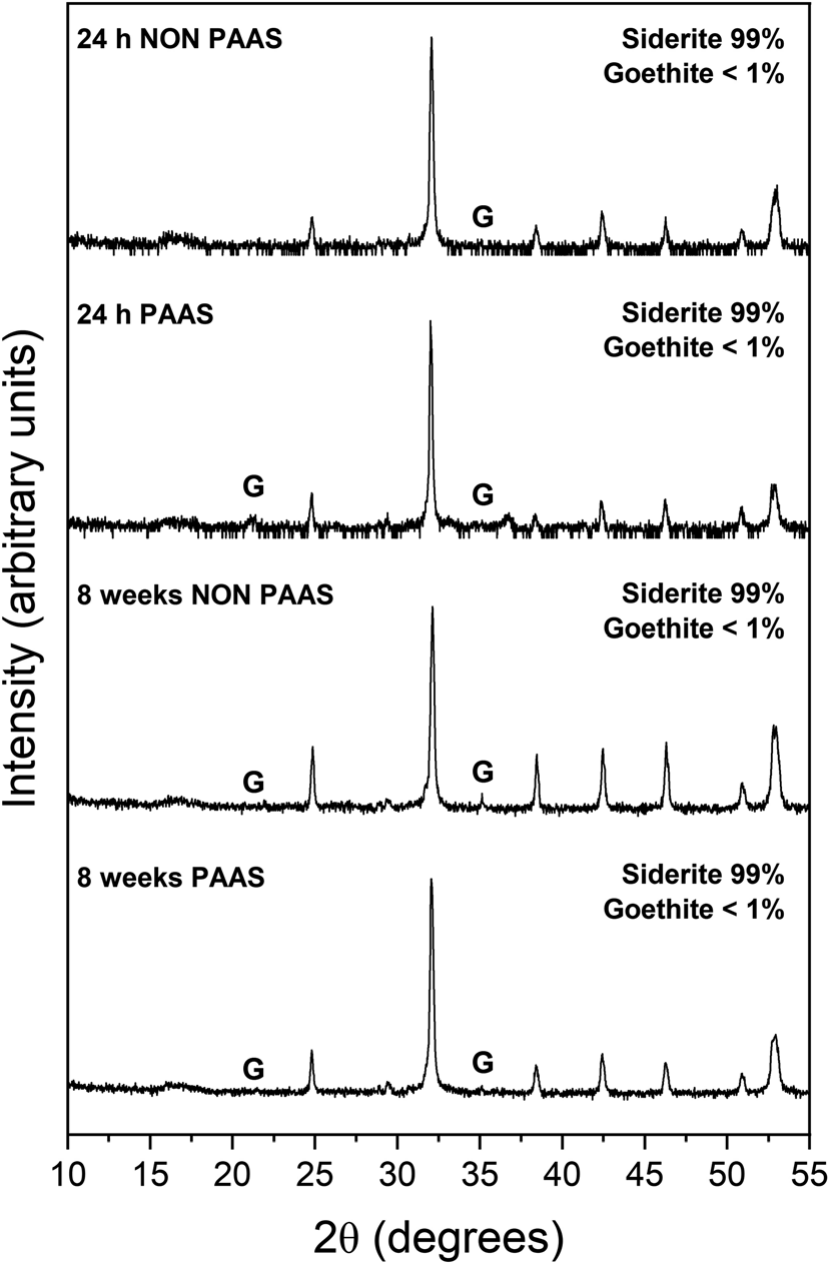
Powder XRD patterns of goethite (G) in the equal concentration (non-PAAS) and PAAS experiments at 50 °C after 24 hours and 8 weeks (Bragg peaks that have not been assigned to any phase correspond to siderite).

The extent of the replacement reaction was found to be time- and temperature-dependent. At the lowest temperature (50 °C), REE-bearing mineral formation was not detected in XRD and only the precipitation of minor (<1%) amounts of goethite, below the quantification level of XRD, was recorded after 56 days (Fig. 1). At higher temperatures, the extent and rate of siderite host replacement depended on the REE ratio in the aqueous solution, with faster replacement occurring in experiments using PAAS solutions: At 165 °C in the equal concentration solution, 40% of the siderite was consumed after 24 hours, compared to 70% in the PAAS solution (Table 1). In the 205 °C experiments, full replacement was observed after 1 week in the equal concentration experiments, compared to less than 24 hours in the PAAS solution experiments.

**Fig. 2:**
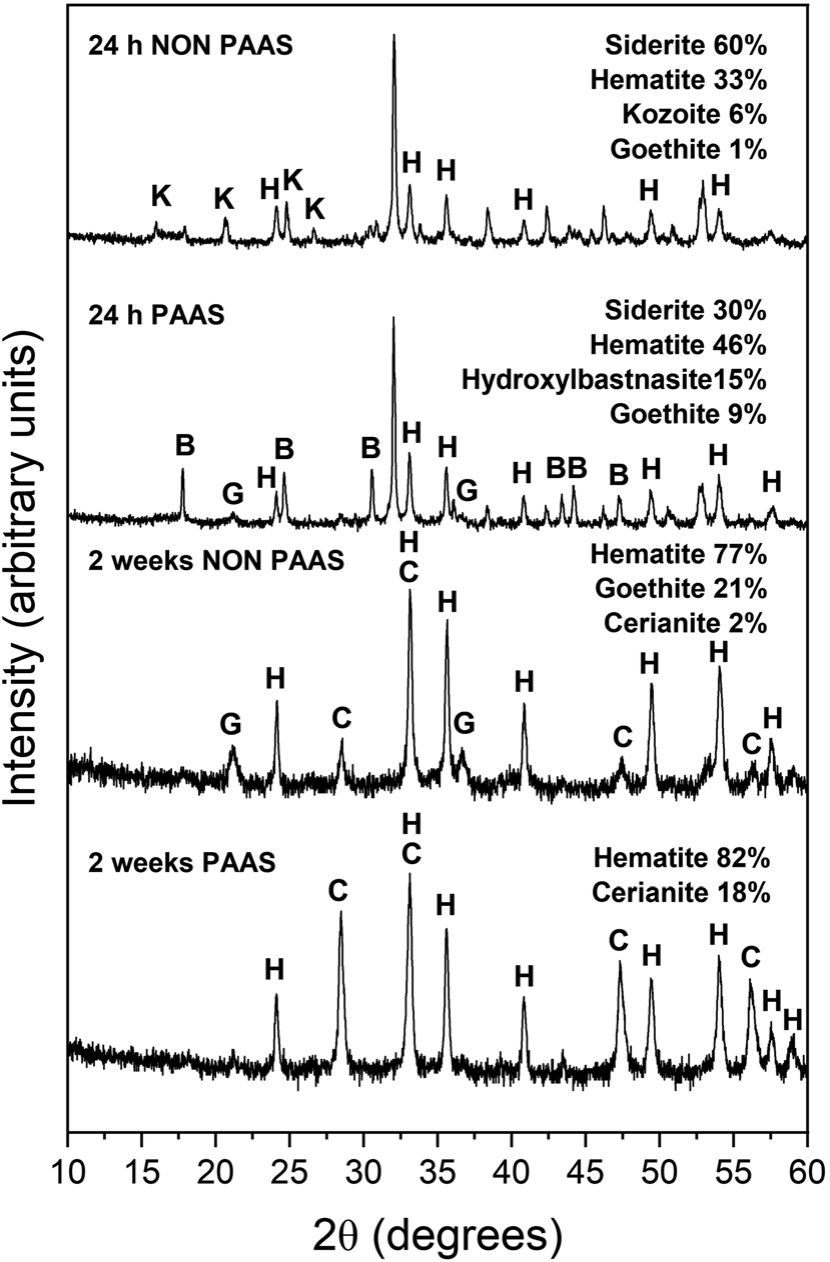
Powder XRD patterns of goethite (G), hematite (H), kozoite (K), hydroxylbastnasite (B) and cerianite (C) in the equal concentration (non-PAAS) and PAAS experiments at 165 °C after 24 hours and 2 weeks (Bragg peaks that have not been assigned to any phase correspond to siderite).

**Fig. 3:**
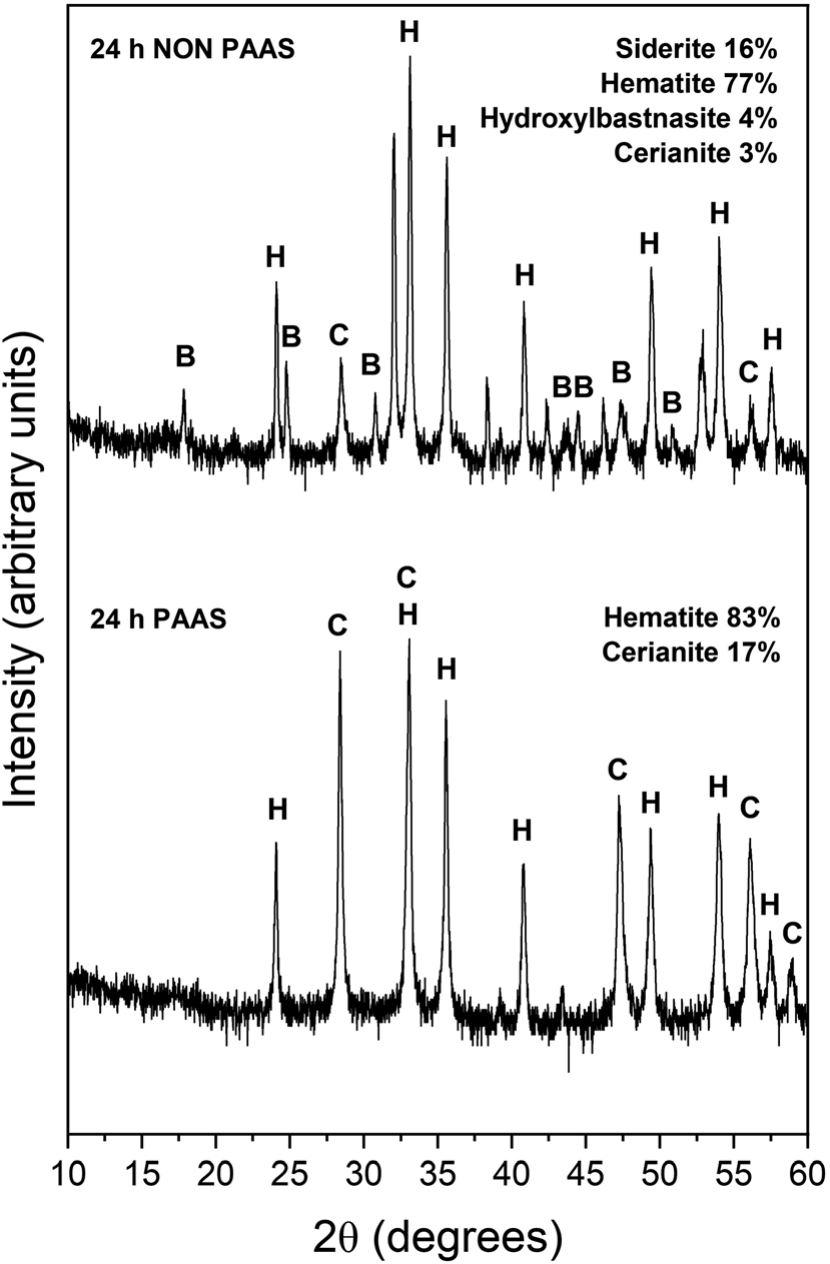
Powder XRD patterns of, hematite (H), hydroxylbastnasite (B) and cerianite (C) in the equal concentration (non-PAAS) and PAAS experiments at 205 °C after 24 hours (Bragg peaks that have not been assigned to any phase correspond to siderite).

The observed mineralogy was also temperature- and REE ratio-dependent: At 165 °C, a rapid transformation (<24 hours) of goethite to hematite was observed, accompanied by the formation of the rare earth carbonates kozoite and hydroxylbastnasite (Fig. 2). After 14 days in equal concentration solutions and 7 days in PAAS solutions, a secondary replacement of the rare earth carbonates by cerianite was observed. In both the 165 and 205 °C experiments, siderite was fully replaced by hematite and cerianite after 7 days (Fig. 3). While in the equal concentration experiments, not all of the goethite transformed to hematite, in the PAAS solution, hematite and cerianite were the final phases formed. In both 165 and 205 °C experiments, the final amount of cerianite in the system was also higher in the PAAS solution experiments (18% compared to 2%, at 165 °C).

### Scanning electron microscopy-energy dispersive spectroscopy (SEM-EDS)

**Fig. 4:**
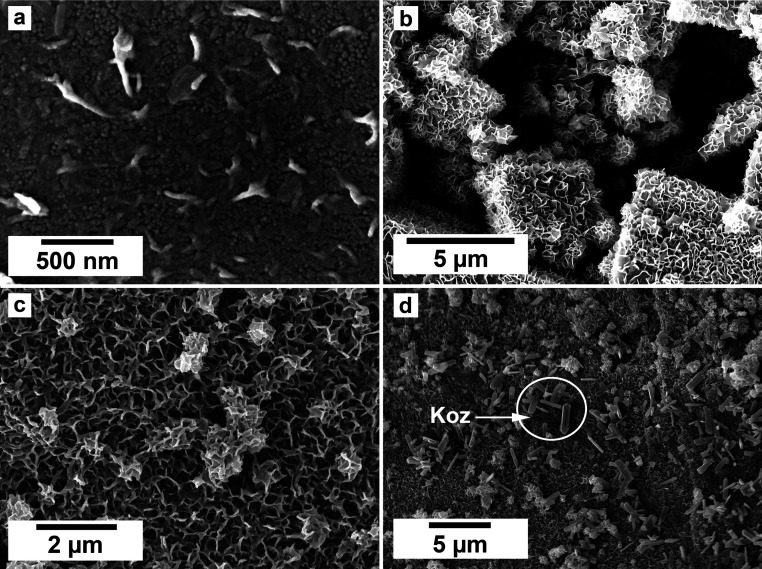
The gradual and complete covering of siderite by a thin (<2 μm) layer of goethite at 50 °C after (a) 48 hours, equal concentration experiments, (b) 8 weeks, PAAS experiments, (c) 12 weeks, PAAS experiments, and (d) goethite and kozoite (koz) crystals after 12 weeks in equal concentration experiments.

**Fig. 5:**
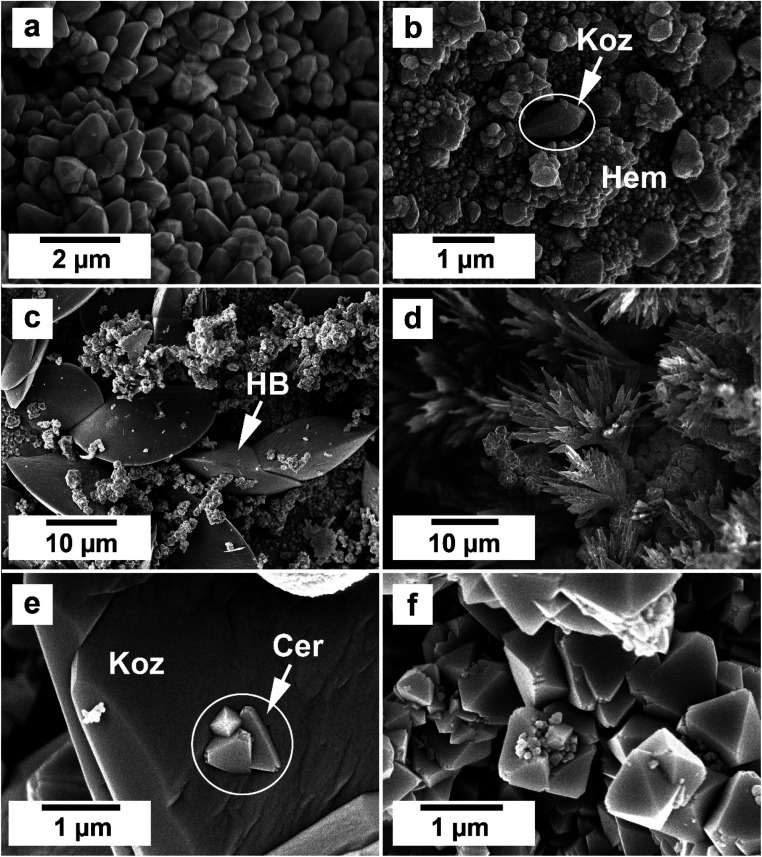
(a) Hematite prisms covering the surface of siderite in equal concentration at 165 °C after 1 week (b) kozoite prism on hematite in equal concentration solution at 165 °C after 24 hours; (c) hydroxylbastnasite in a matrix of ceriante and hematite in equal concentration solution at 165 °C after 24 hours; (d) hydroxylbastnasite dendrites in PAAS solution 165 °C after 24 hours; (e) cerianite growing on kozoite surface in equal concentration solution at 205 °C after 24 hours; (f) cerianite prisms in equal concentration solution at 205 °C after 1 week.

SEM imaging revealed the formation of precipitates on the surface of the siderite grains. The extent to which the newly formed precipitates covered and replaced the host grain was found to be time- and temperature-dependent. For example, at 50 °C, the surface of the siderite grains became fully covered by a thin layer (<2 μm) of goethite nanocrystals with flaky morphologies (Fig. 4). Minor amounts of kozoite with similar morphologies reported by Szucs *et al.* (2021),^35^ Szucs *et al.* (2022)^36^ and Maddin *et al.* (2024)^37^ were also observed in images taken after 12 weeks in the equal concentration experiments (Fig. 4d), however, the amount was too small to be detected by XRD. At 165 °C, the newly formed phases consisted of triangular prisms of hydroxylbastnasite, pseudohexagonal prisms of hematite, and cubes and bipyramidal prisms of cerianite (Fig. 5).

**Fig. 6:**
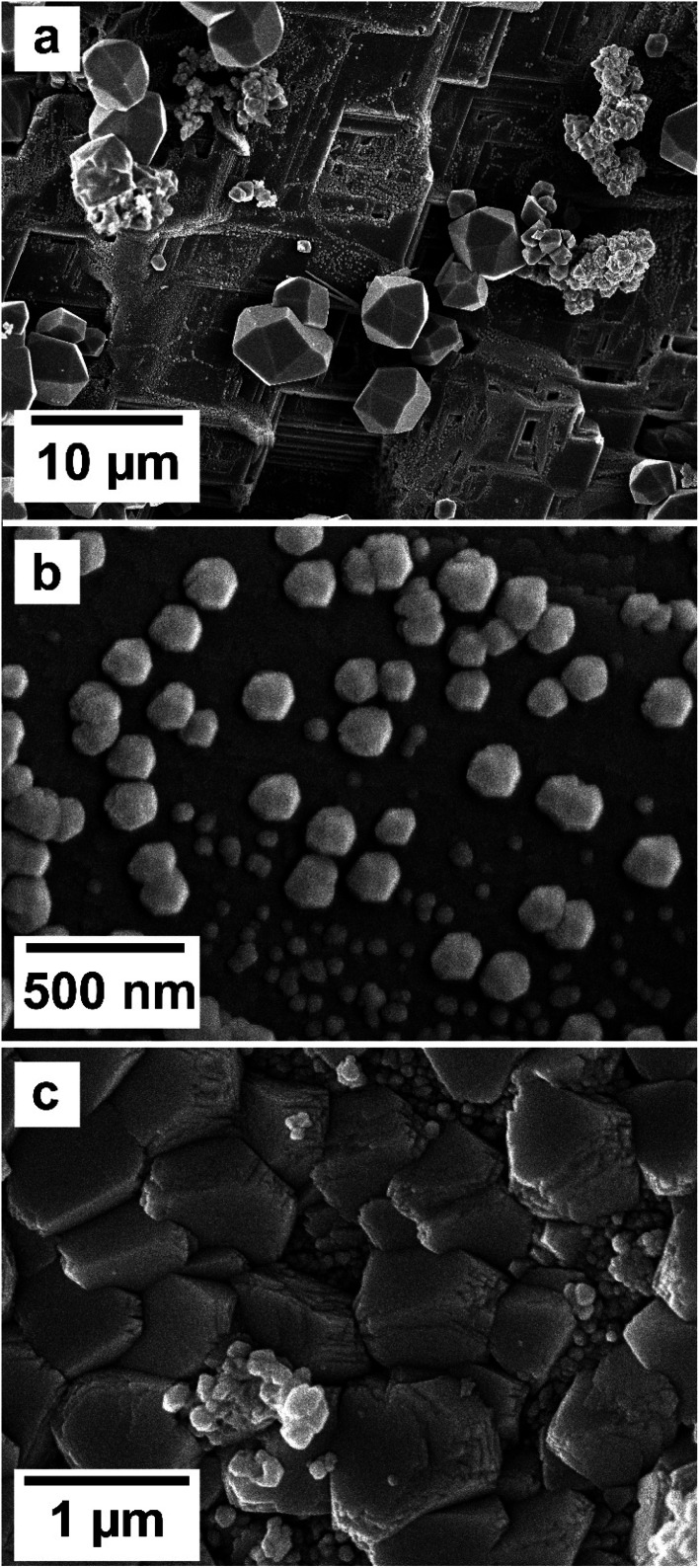
Growth of hematite on siderite and epitaxial growth. (a) Hematite growing on the surface of siderite, forming μm-sized and nanocrystals on the surface of the host in equal concentration solution at 205 °C after 24 hours. (b) Hematite growing oriented on the surface of siderite in equal concentration solution at 205 °C after 24 hours. (c) μm-sized hematite coating the surface of siderite, showing a loss of their initial non-random orientation in equal concentration solution at 165 °C after 1 week.

**Fig. 7:**
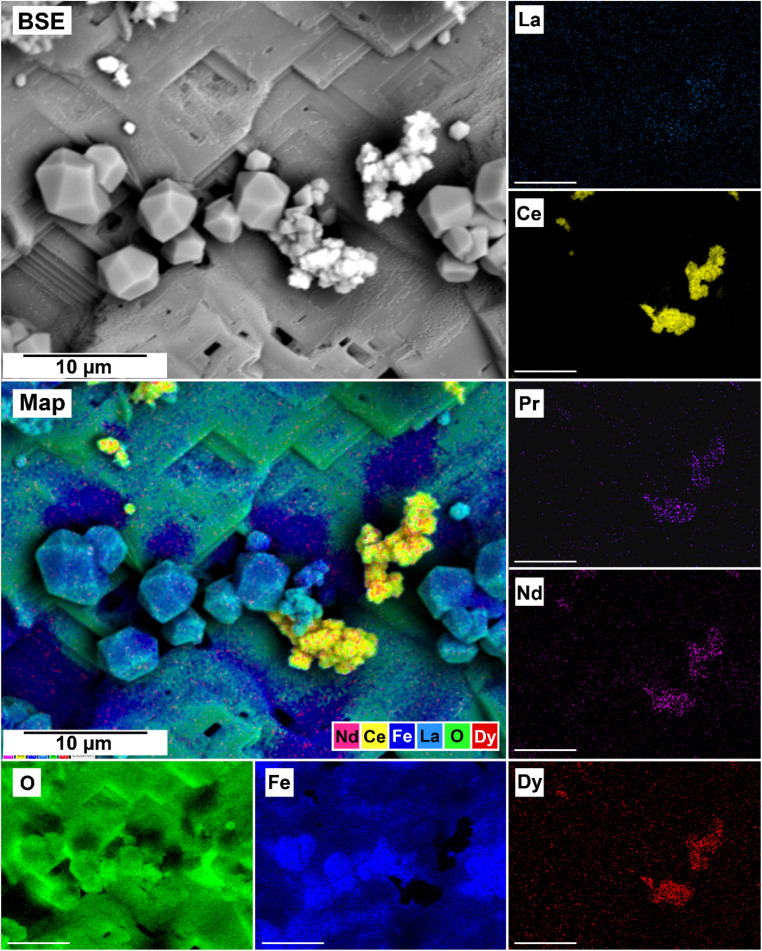
Breakdown of siderite and subsequent detachment of the newly formed hematite and cerianite crystals in equal concentration experiments at 205 °C after 24 hours.

**Fig. 8:**
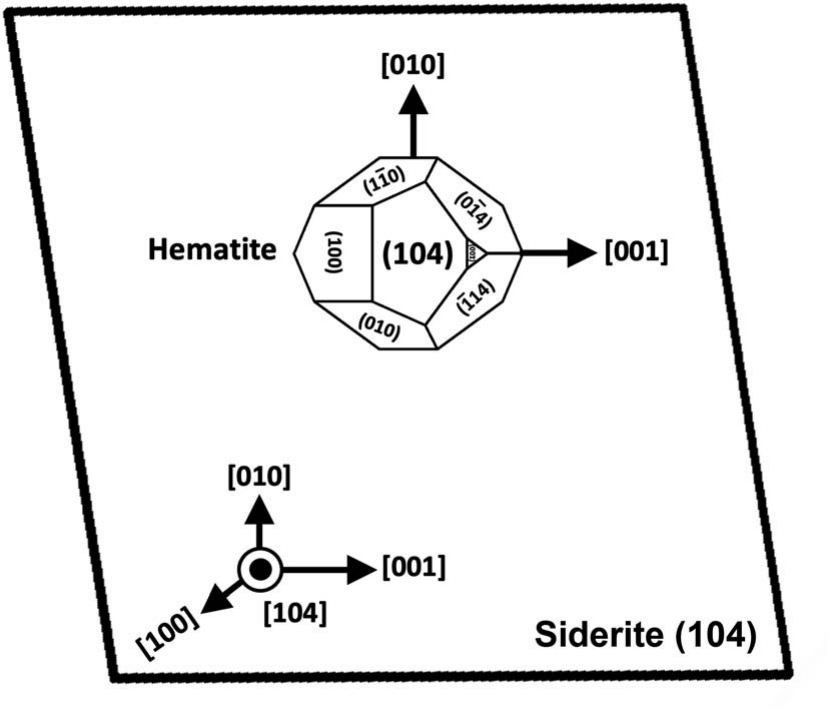
Schematic diagram showing the epitaxial relationship between hematite and siderite crystals.

SEM imaging also revealed that the early stages of hematite crystallization on the siderite surfaces were characterized by the formation of prismatic nanocrystals with diameters of ∼150–200 nm (Fig. 6a and 7). Many of these nanocrystals exhibited a transient, non-random orientation on the (104) face of siderite, indicative of epitaxial overgrowth (Fig. 8). Specifically, this hematite–siderite epitaxial relationship was defined by the parallel alignment of the crystallographic axes of the hematite to those of the underlying siderite substrate (Fig. 6b). However, once the hematite crystals grew and coalesced, this orientation was lost (Fig. 6c).

**Fig. 9:**
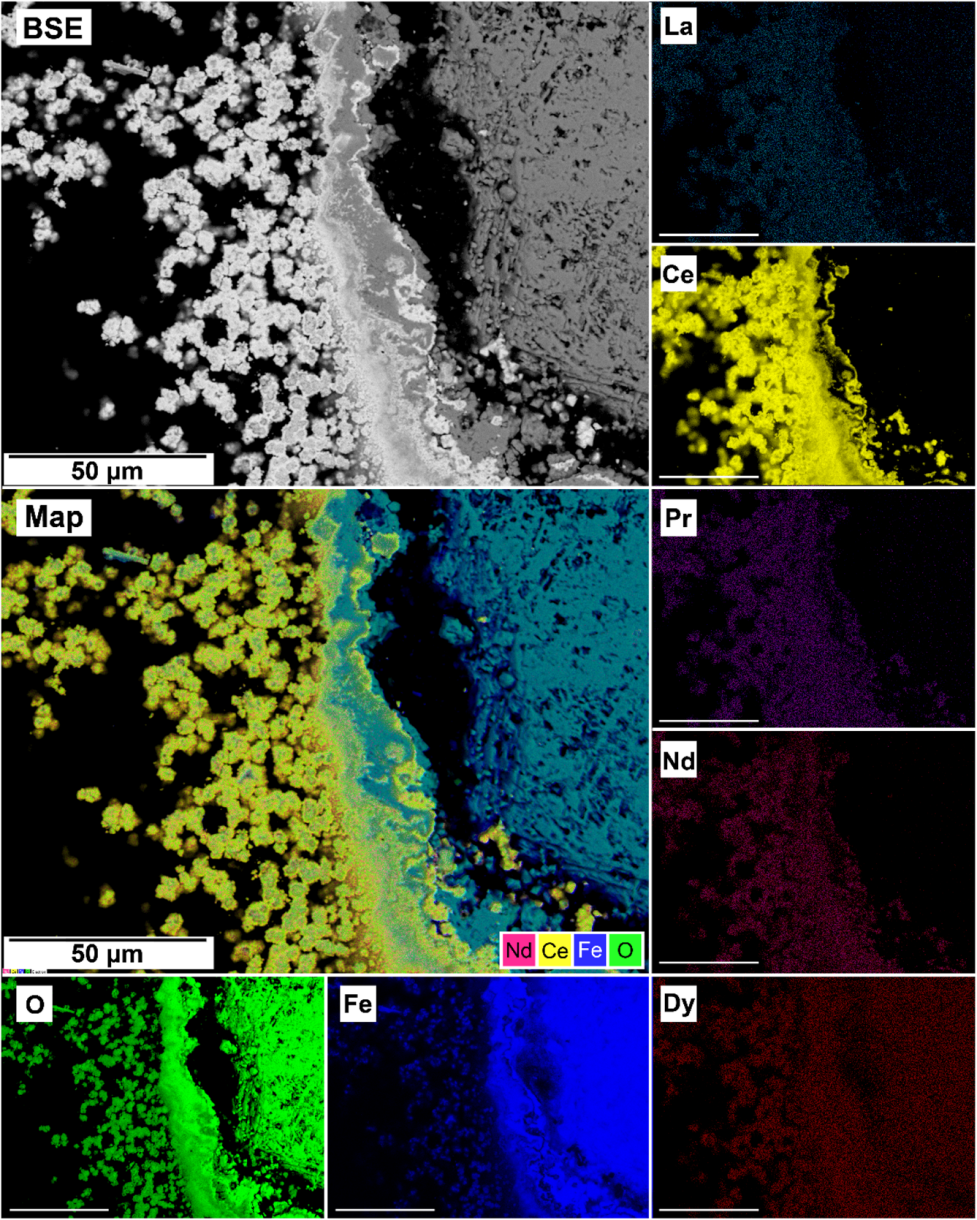
SEM-BSE image of siderite fully replaced by hematite and cerianite at 205 °C after 1 week in equal concentration experiments and EDS maps of La, Ce, Pr, Nd, Dy, O, and Fe showing the homogenous distribution.

**Fig. 10:**
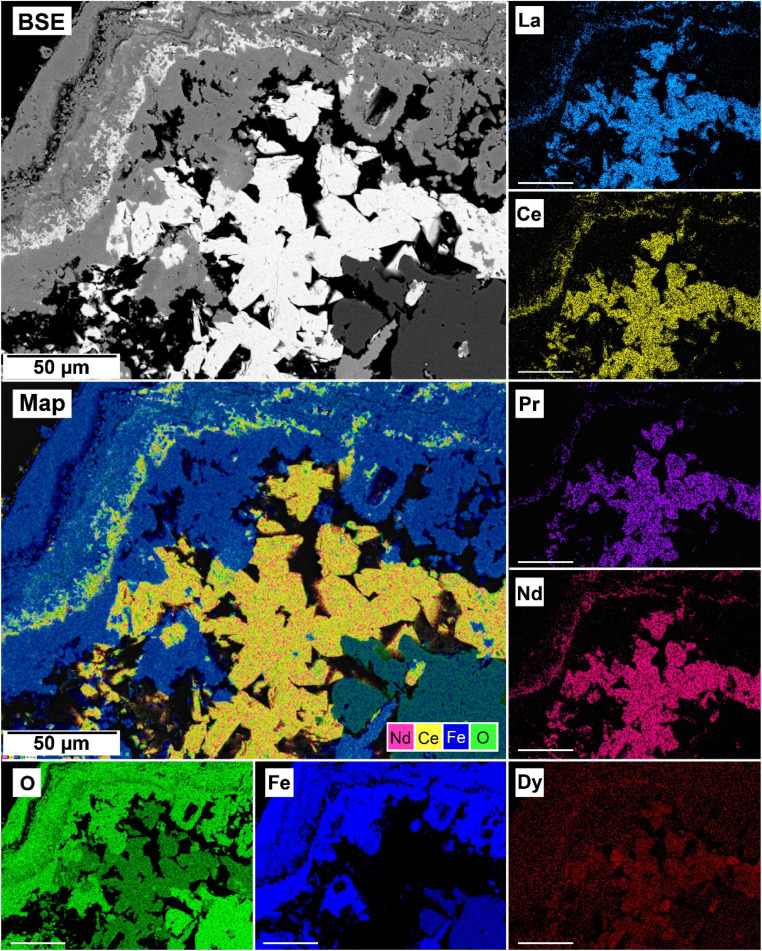
Partial replacement of the siderite host with kozoite in equal concentration experiments at 165 °C after 24 hours.

**Fig. 11:**
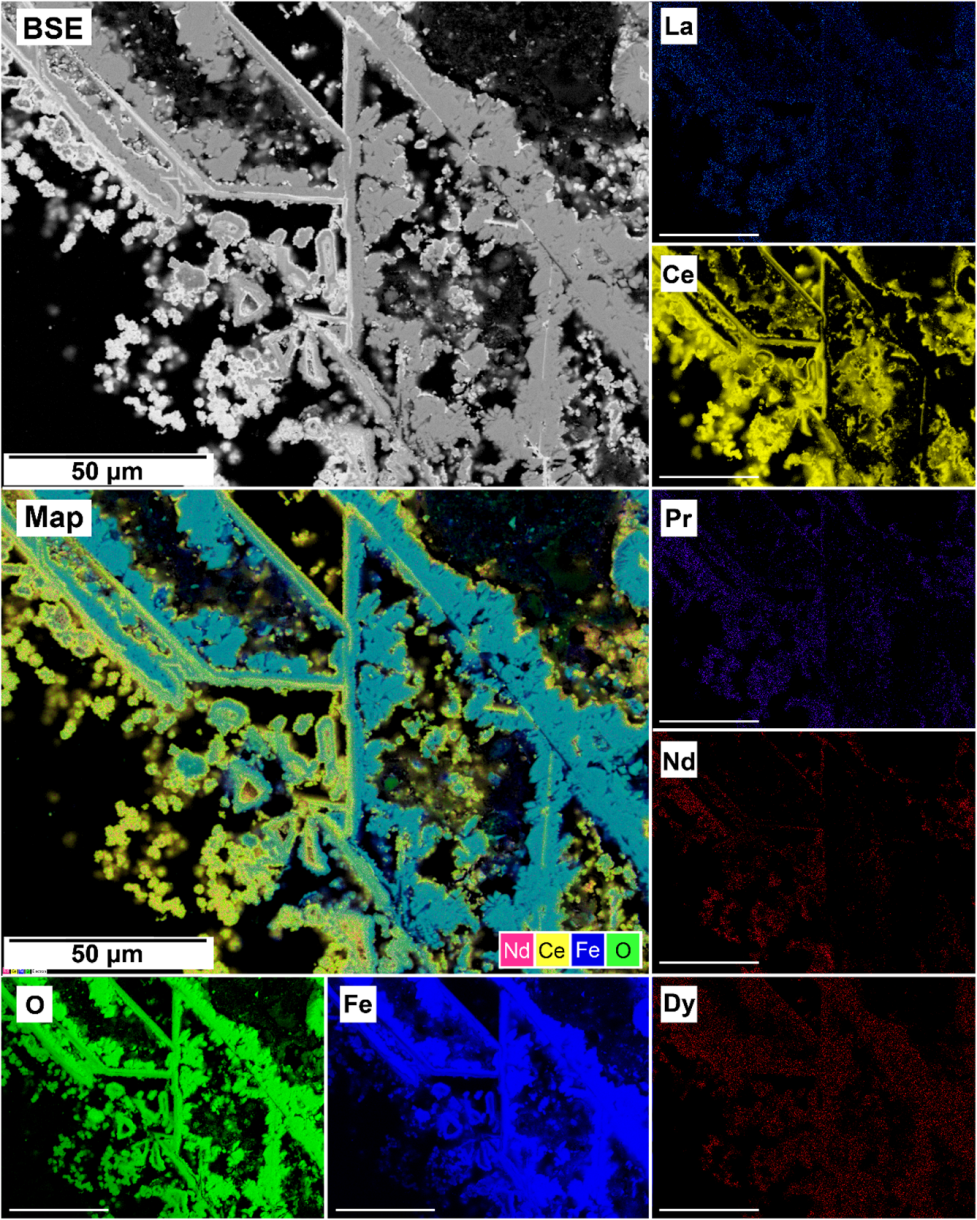
SEM-BSE image of siderite grain fully replaced by hematite and cerianite at 205 °C after 24 hours in the PAAS experiments and EDS maps of La, Ce, Pr, Nd, Dy, O, and Fe showing the heterogenous distribution.

**Fig. 12:**
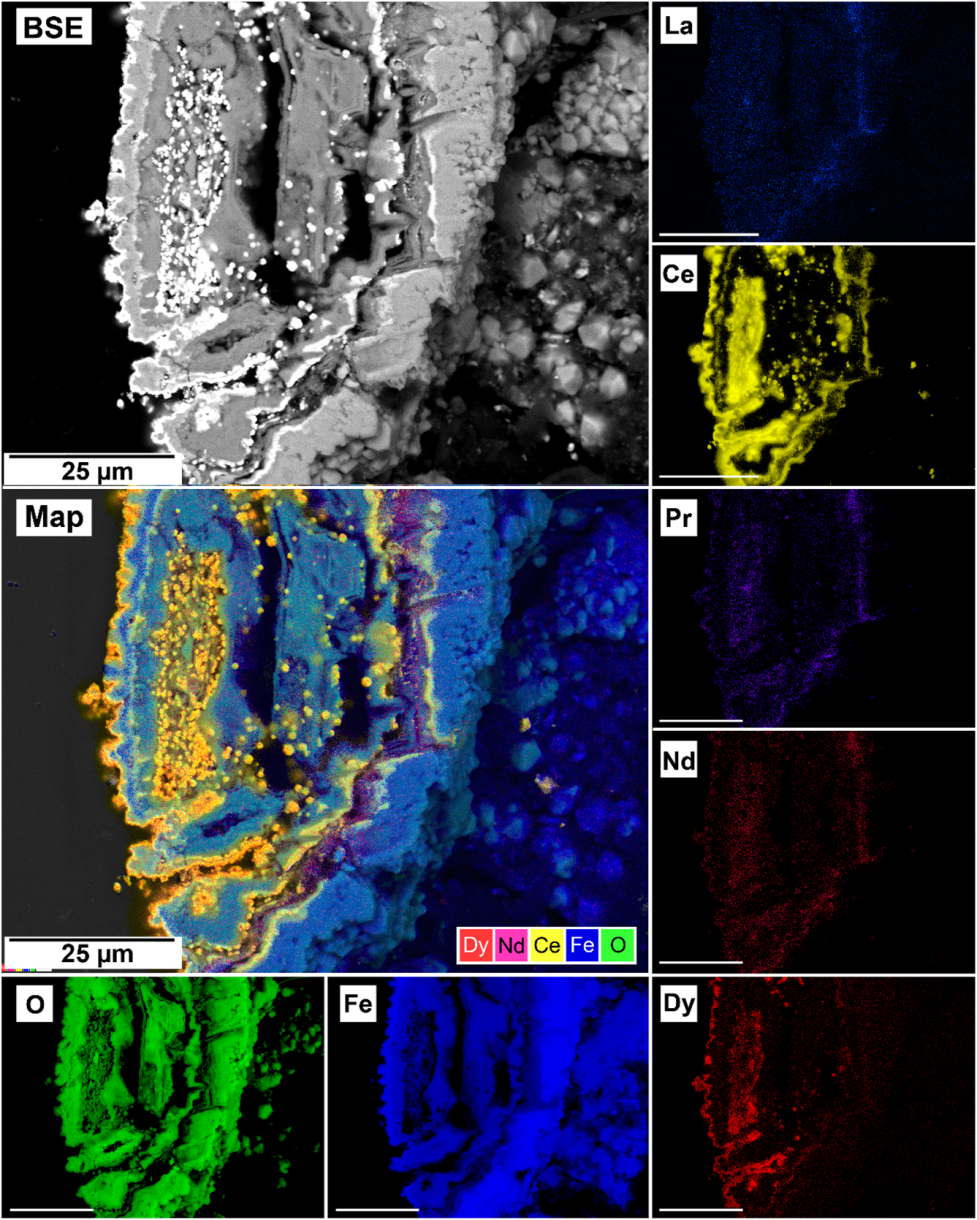
Selected grain of siderite from an equal concentration experiments at 205 °C after 24 hours incorporation of Dy in cerianite as impurities.

**Fig. 13:**
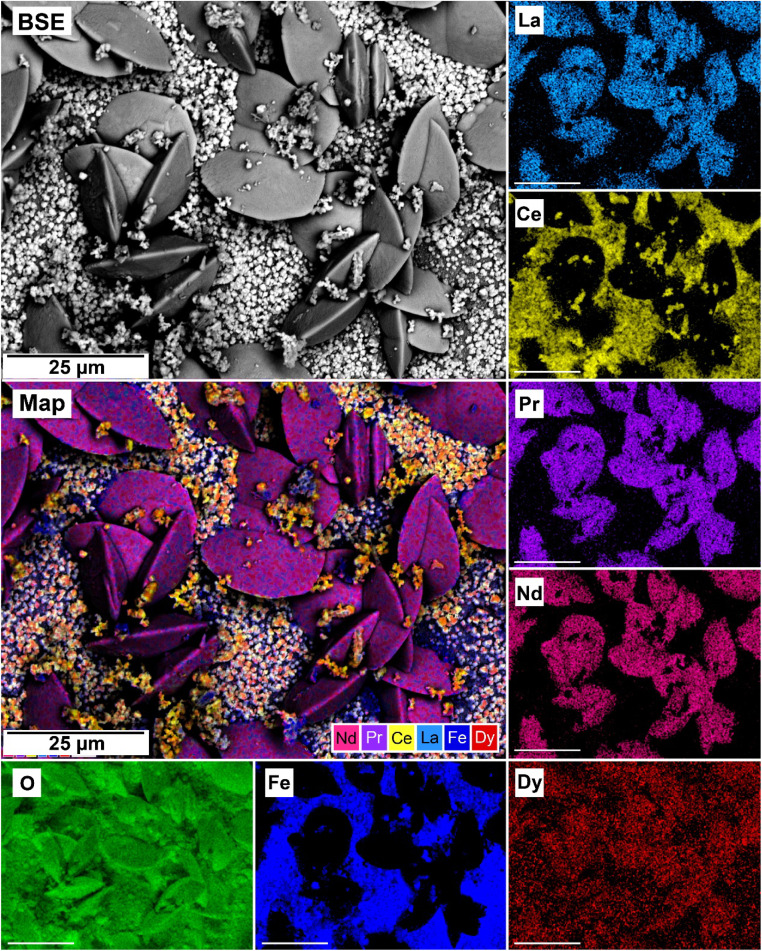
SEM-BSE image of siderite grain being replaced with bastnasite in the equal concentration experiments at 205 °C after 24 hours and EDS maps showing the almost homogenous distribution of REE.

**Fig. 14:**
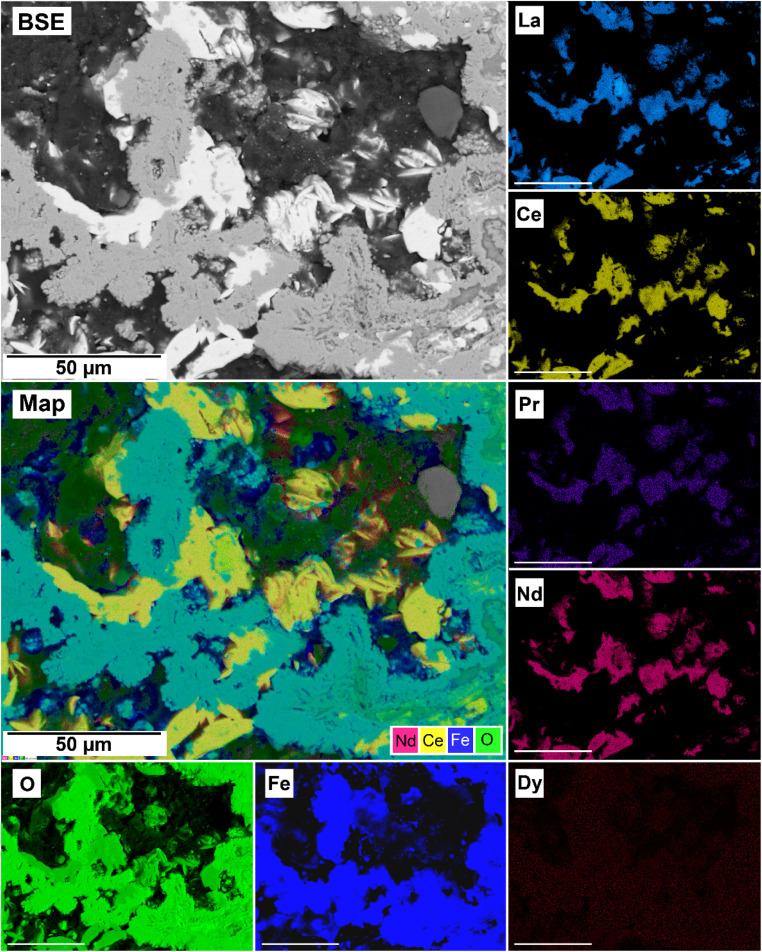
SEM-BSE image of siderite grain being replaced with kozoite in PAAS solution at 165 °C after 24 hours and EDS maps showing the almost heterogenous distribution of REE.

The EDS analyses of the cross section of the grains in the polished epoxy resin pucks revealed differences in spatial distribution of the REEs in the newly formed solids. The chemistry of the REE-bearing minerals was found to be dependent on the REE ratio in the initial aqueous solution, with a more homogenous distribution in the equal concentration solution (Fig. 9 and 10) and a more heterogenous distribution in the PAAS solution (Fig. 11). A preferential uptake of Nd and La with Ce in the PAAS solution was observed at 165 °C after 24 hours (SI-1 Line Analysis, ESI†), as well as Dy incorporation into the cerianite in the PAAS solution at 205 °C after 1 week (SI-2 ESI†), and in the equal concentration at 205 °C after 24 hours (Fig. 12). In general, the composition and ratio of the REE in the bastnasite and kozoite appeared to record the composition of the original fluid phase (equal concentration and PAAS experiments; Fig. 13 and 14), with almost equal uptake of REEs in bastnasite (Fig. 10) and preferential uptake of La, Ce and Nd in kozoite (Fig. 11). In contrast, REE incorporation in the iron oxides was below the SEM-EDS detection limit, at <0.1%. (SI-3 ESI,† point analysis).

## Discussion

The interaction of siderite with our REE-bearing aqueous solutions resulted in the initial dissolution of the host grain, releasing Fe^2+^ and CO_3_^2−^ ions into the aqueous solution. As temperature increased, siderite broke down^38^ and the aqueous Fe^2+^ ions then became oxidized and precipitated as iron oxides goethite and hematite. The CO_3_^2−^ interacted with the REE in solution leading to the crystallisation of rare earth minerals kozoite and hydroxybastnaesite, which then subsequently transformed into the oxide cerianite due to the oxidation of Ce^3+^. The resulting textures and mineralogy are consistent with the solvent-mediated surface precipitation and subsequent pseudomorphic mineral replacement produced by the interaction of the hydrothermal fluids with the primary siderite.

### The formation of iron oxides as a function of temperature and pH

Siderite is a ferrous mineral found in oxygen-free environments, such as lakes, rivers, and marine sediments.^39–41^ Its stability, dissolution, and the subsequent precipitation of iron oxides are temperature- and pH-dependent processes that significantly influence the kinetics of siderite breakdown.^42–44^ Ferrous iron in aqueous solutions readily reacts with dissolved oxygen, oxidizing to ferric iron (Fe^3+^) and precipitating as iron oxides and/or oxyhydroxides such as goethite, hematite, magnetite, maghemite, and ferrihydrite.^39,45,46^

Many studies have shown that a poorly-ordered nanoparticulate phase, two-line ferrihydrite, is a predominant precursor to several iron oxides.^47–49^ Under oxic conditions, two-line ferrihydrite gradually transforms to more thermodynamically stable phases, such as goethite and hematite.^47,49^ It was Bohm (1925)^50^ who first demonstrated that an “amorphous Fe(iii) hydroxide” rapidly transformed to pure goethite when maintained under 2 M KOH at 150 °C for 2 hours, whereas hematite was the main end product if the material was subjected to hydrothermal conditions.^48^ This demonstrated that this ferrihydrite–goethite transformation is highly temperature- and pH-dependent. The rate of transformation of ferrihydrite is relatively slow at ambient temperature. For example, Schwertmann & Murad^48^ recorded 19% of the initial concentration of ferrihydrite remained after 970 days at pH ≤ 6. However, at a temperature of 92 °C, crystallization occurs faster, with hematite formation beginning within 10 minutes and a complete transformation occurring after 116 hours. Our experiments did not reveal the presence of ferrihydrite in the XRD patterns. However, we cannot fully disregard the possibility of its formation. Due to the rapidity of its transformation in the temperature and pH parameters of our experiments, we cannot discard the possibility that it may have been present as a transient phase during the very early stages of the reaction, quicky crystallising to the more thermodynamically stable goethite and hematite.

In our experiments, temperature significantly influenced the oxidation processes of siderite, affecting the kinetics, polymorph selection, and formation of chemical textures during the replacement reactions due to increased dissolution rates and differences in solubilities. At the lowest temperature of 50 °C, we recorded the initial dissolution of siderite and the crystallization of a thin layer of goethite that covered the entire surface of the siderite grain (Fig. 4). However, the transformation did not progress further once the siderite surface had been covered by goethite. Our PHREEQC calculations revealed that even during the earliest stages of dissolution of siderite (SI_siderite_ = −15), the aqueous solution can become supersaturated in goethite (SI_goethite_ = +2.78), showing that the dissolution of just a few monolayers of the siderite surface would result in a fluid boundary layer that would allow the crystallisation of this iron oxide. We propose that this coating of goethite on the siderite grains isolated the host from the aqueous solution, halting any further dissolution of siderite, and promoting a state of partial equilibrium^36,51,52^ which inhibited further recrystallisation and formation of REE carbonates. A similar result was observed by Yu *et al.*, 2023, 2024 who investigated the effects of dissolved oxygen (DO) concentrations on As(v) adsorption onto siderite. They found that while As(v) adsorption capacities increased with an increase in DO concentration, excess DO caused fast oxidation of siderite, constraining As(v) adsorption. This was due to the production of a coating of Fe(iii) oxides on the surface of the pristine siderite that prevented any further oxidation of the host.^53,54^

At 165 °C we observed an increase in the amount of goethite precipitation as well as its subsequent transformation to hematite (Table 1). Parallel to the Fe^2+^ oxidation processes we also observed the crystallisation of REE carbonates. The transformation of goethite to hematite is strongly influenced by temperature and pH.^55–58^ It is known that the transformation from goethite to hematite can occur at temperatures ranging from 160–300 °C.^48,57,59^ There is also a relationship between temperature and pH, with faster transformation rates at higher temperatures coinciding with higher pH. For example, Das *et al.* (2010)^47^ reported the formation of goethite and hematite at 50 °C and pH 2 after 7 days, while similar peak intensities were evident on after 5 days at pH 7 and after only after 21 hours at pH 10. Concomitantly, the transformation of goethite to hematite was also observed with extended reaction time. Hematite formation was also promoted at near neutral pH conditions, while goethite formation was favour acidic (∼4) or highly alkaline (∼12) conditions.^47,48^ This is consistent with the evolution of our experiments: Our REE solution had an initial pH of ∼5.5, promoting the formation of goethite. As the siderite dissolved and REE carbonates formed, the pH gradually became more basic, moving towards a pH of ∼8.2, promoting the transformation from goethite to hematite.

The solubility of siderite and the rate of its dissolution are highly dependent on temperature and pH. Bénézeth *et al.*, 2009 (ref. 60) investigated the solubility of natural siderite from 25 to 250 °C. Their experimental work showed that the values for log_10_ *K*_sp-siderite_ decrease with increasing temperature. For example, at 50 °C log_10_ *K*_sp-siderite_ = −10.19 compared to log_10_ *K*_sp-siderite_ = −12.19 at 200 °C. Concomitantly, and similar to other divalent cation carbonates, the dissolution rate of siderite decreases with an increase in pH. Golubev *et al.* (2009)^38^ experimentally demonstrated that this decrease follows a linear trend, spanning approximately 1.5 orders of magnitude between pH 1 and 5. Furthermore, the dissolution rate is strongly temperature-dependent, exhibiting a variation of approximately 2 orders of magnitude between 25 and 100 °C. Taking the initial pH of the REE-bearing aqueous solution (5.1) into consideration, at 50 °C the solubility of siderite would be ∼10^−10.0^–10^−11.1^ mol cm^−2^ s^−1^ and at 100 °C the value would be ∼10^−10.0^–10^−9.9^ mol cm^−2^ s^−1^. Although no dissolution rates have been reported for temperatures above 100 °C, the observed trend suggests that the dissolution rate would increase ∼1.5–2.0 orders of magnitude at 200 °C.

At low pH values, particularly in acidic conditions (pH < 5), the dissolution of siderite is promoted due to the increased activity of hydrogen ions in the solution. The adsorption of H^+^ onto the mineral surface enhances the dissolution process, leading to higher concentrations of dissolved Fe^2+^ in the aqueous phase.^61^ Studies have shown that siderite dissolution rates increase significantly at pH levels around 2 to 5, where the mineral becomes more reactive.^61^ This increased dissolution can facilitate the release of Fe ions, which can subsequently precipitate as iron oxides such as magnetite or hematite, depending on the redox conditions present.^62^

Conversely, at neutral to alkaline pH levels (pH ≥ 7), the solubility of siderite decreases, resulting in slower dissolution rates. In circumneutral conditions, the stability of siderite is enhanced, making it more resistant to dissolution.^63^ In environments with circumneutral pH, siderite can persist due to the buffering capacity of the surrounding media, which limits the availability of H^+^ ions necessary for dissolution.^63^ This resistance to dissolution can hinder the formation of iron oxides and REE-bearing minerals, as the necessary precursor ions may not be sufficiently available in the solution.

Furthermore, carbonate species HCO_3_^−^ and CO_3_^2−^ can also influence the dissolution kinetics of siderite. These species can act as inhibitors of dissolution at higher pH levels, as they compete with H^+^ ions for adsorption sites on the mineral surface.^64^ This competition can further reduce the rate of siderite dissolution and, consequently, the availability of iron for subsequent mineral formation.

The formation of REE-bearing minerals is also pH-dependent. In acidic conditions, the increased solubility of siderite can lead to higher concentrations of Fe^2+^ in solution. For example, the presence of dissolved REE in acidic brines can facilitate their incorporation into iron oxides precipitating from the dissolved siderite.^65^ However, in neutral to alkaline conditions, the reduced dissolution of siderite limits the availability of Fe^2+^, thereby slowing the formation of the iron oxide minerals.

### Siderite–hematite epitaxial overgrowth

**Fig. 15:**
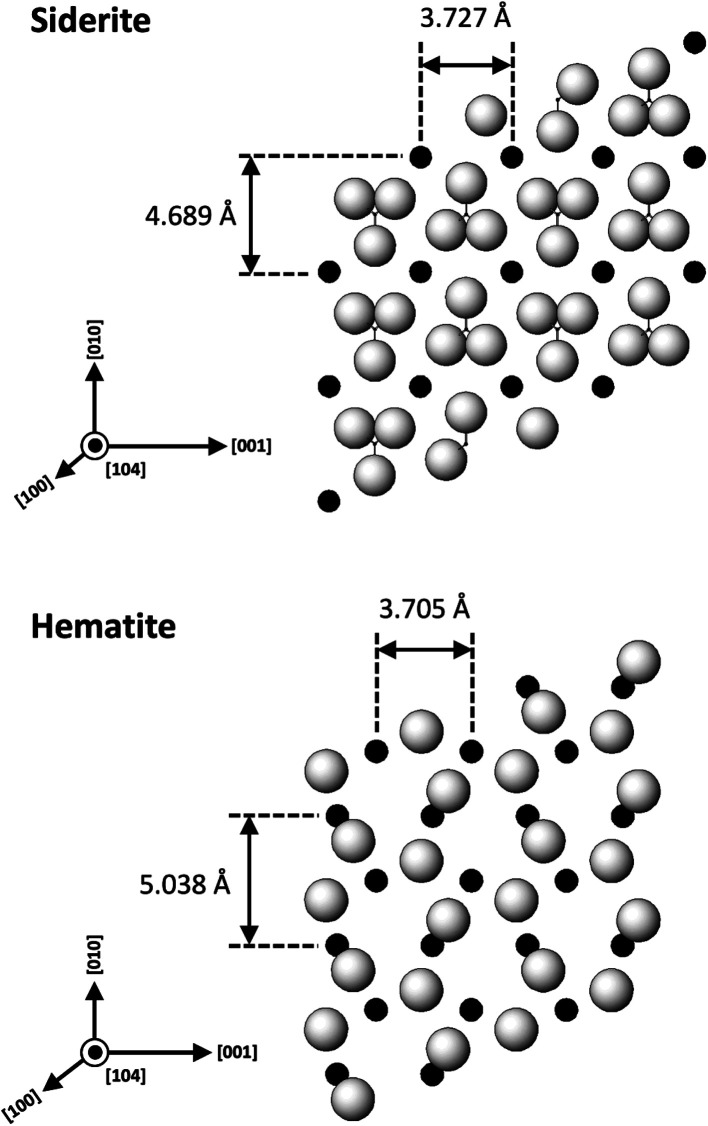
Projection of the atomic structures of a (104) slices of hematite and siderite showing the main directions of epitaxy and repeating periods.

An interesting feature observed in the 165 °C experiments was the presence of oriented overgrowths of the newly formed hematite on the surface of siderite (Fig. 6). This epitaxial overgrowth is not unexpected, given that hematite and siderite share the same crystal system (trigonal) and space group (*R*3̄*c*). Siderite has lattice parameters of *a* = *b* = 4.6916 Å, and *c* = 15.3796 Å,^66^ while hematite lattice parameters are *a* = 5.038 Å and *c* = 13.772 Å.^67^ These structural similarities suggest a favourable fit for epitaxial growth (Fig. 15). The misfit values have been calculated using the expression:^68^
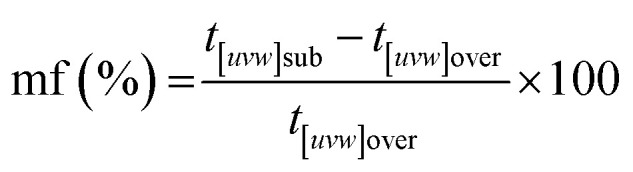
These can be used to quantify the degree of lattice mismatch between the epitaxial overgrowth and the substrate. In this expression, *t*_[*uvw*]sub_ and *t*_[*uvw*]over_ correspond to the repeating periods along the matching directions [*uvw*] of the substrate (siderite) and the overgrowth (hematite), respectively (van der Merwe, 1978).^69^

The calculated misfit values for the (104)_sid_[010]_sid_‖(104)_hem_[010]_hem_ and the (104)_sid_[001]_sid_‖(104)_hem_[001]_hem_ alignments are −6.93 and +0.59% respectively, falling well below the generally accepted limits (15–20%) required for epitaxial overgrowth to occur.^70^ The negative value indicates that the unit cell of hematite is very slightly contracted along the [010] direction of epitaxy of siderite, while the small positive value shows an almost perfect match (with a slight expansion) of the unit cell of goethite with respect to the siderite in the [001] direction.

As hematite forms as a consequence of the hydrothermal alteration and Fe^2+^ to Fe^3+^ oxidation process, there are two factors that need to be taken into account: first is the slightly larger molar volume of hematite (30.39 cm^3^ mol^−1^)^71^ compared to siderite (29.33 cm^3^ mol^−1^),^71^ which is likely contributing to the loss of orientation once the hematite crystals grow and coalesce. Second is the dissolution and breakdown of siderite due to the hydrothermal replacement by REE carbonates and the Fe^2+^ to Fe^3+^ oxidation process, contributing to the subsequent detachment of the newly formed hematite crystals from the host (Fig. 7). However, it is important to consider that although the ideal composition of pure siderite is FeCO_3_, this mineral is an end member of a complex solid solution (CaCO_3_–MgCO_3_–FeCO_3_) and therefore can contain other divalent ions such as Ca^2+^, Mg^2+^ or Mn^2+^, alongside impurities such as Zn^2+^, Co^2+^, or Cd^2+^.^72^ These chemical variations have the potential to influence the matching unit cells and subsequently impact the kinetics of siderite replacement processes, as well as the final solid morphology of hematite.

### Formation of rare earth carbonates and their secondary replacement by cerianite

Our 165 °C experiments resulted in the partial replacement of the siderite host with the rare earth carbonates kozoite and hydroxylbastnasite (Fig. 10 and 13). The reaction sequence for REE carbonates was found to be time-and temperature-dependent,^37^ with the initial formation of kozoite between 50 and 165 °C and its subsequent transformation to the more stable hydroxylbastnasite at temperatures ≥165 °C. The rate of crystallization and composition of the REE-bearing phases is known to be controlled by the solubilities and dissolution rates of the host grain, the REE ratio in solution, as well as the evolving fluid composition during the replacement process.^37^ The dissolution of siderite and its subsequent replacement from the periphery inwards was a consequence of the lower solubilities of REE carbonates compared to the siderite host. The solubility products for pure Nd-hydroxylbastnasite and Nd-kozoite have been calculated to be log(*K*_sp_) = −23.8 ± 0.1 and log(*K*_sp_) = −22.3 ± 0.2, respectively,^34^ while the solubility product of siderite, log(*K*_sp_) = −10.94 ± 0.4 (ref. 56) is much higher. Our PHREEQC calculations show that high supersaturation levels for kozoite and bastnasite (SI > 1–3) as well as cerianite (SI > 11) are reached when the REE-bearing solutions (equal concentration or PAAS) are equilibrated with siderite at 165–205 °C, also even if the solution has not fully reached saturation for the host. Siderite dissolution rates have also been shown to increase with temperature.^38^ Besides, the siderite transformation to hematite due to the Fe^2+^ to Fe^3+^ oxidation,2FeCO_3(s)_ + 4H_2_O → Fe_2_O_3(s)_ + 2CO_2(g)_ + 8H^+^ + e^−^,was translated into the release of CO_2_ and therefore in the gradual acidification of the aqueous solution, which promoted the subsequent dissolution of all carbonates after 1 week in equal concentration experiments and 24 hours in PAAS experiments (Table 1).

Point analysis of the EDS maps showed that the uptake and incorporation of the REE in the newly formed carbonates was dependent on the initial concentration in solution. In the equal concentration experiments, the uptake of REE by the crystallising carbonate occurs in the same ratio as their composition in the aqueous solution, indicating that the crystallising carbonate mirrors the REE composition of the surrounding solution (SI-4†). Conversely, in the PAAS solution experiments, the REE ratio is different compared to the equal concentration, but still reflects the higher concentration of La, Ce, and Nd in the original solution (SI-4†). On one hand, this aligns with the typical composition of the REE carbonates bastnasite and kozoite. Bastnasite has a hexagonal structure that tends to uptake the light REE (La to Nd) which are in a 9-fold coordination with oxygen,^17,73^ with the heavier Dy only entering as impurities. Heavier REE could potentially be incorporated into the bastnasite structure, however, this would require higher pressure conditions that are outside of our experimental range. In contrast, the intermediate phase kozoite is orthorhombic and it can include both lighter and heavier REE,^17^ perfectly accommodating Dy. On the other side, the mirroring of the REE ratios of the initial aqueous solution (equal concentration and PAAS experiments) could be explained by assuming that kozoite and bastnasite are both primary phases forming in their respective reactions and have not formed *via* metastable intermediate precursors (*e.g.*, lanthanite and tengerite; Vallina *et al.*, 2015;^17^ Szucs *et al.*, 2021;^35^ Szucs *et al.*, 2022).^36^ This would also explain why we did not observe any zoning in the REE carbonates as reported in Maddin *et al.*, (2024).^37^ At 205 °C siderite was fully replaced by hematite and the rare earth carbonates by cerianite. The solution-mediated oxidation of Ce^3+^ to Ce^4+^ initiates cerianite, as described by this reaction:Ce_(aq)_^3+^ + 2H_2_O → CeO_2(s)_ + 4H_(aq)_^+^ + e^−^

The oxidation of Ce^3+^ to Ce^4+^ is triggered by several factors. First, Ce^4+^ has the electron structure of a noble gas, making it more stable than Ce^3+^, and the higher temperatures provide the energy needed to facilitate the loss of an electron. Second, as stated earlier, as our system progressed towards equilibrium, the pH would increase from ∼5 towards ∼8.2. The higher concentration of OH^−^ ions allow the formation of Ce-hydroxo-complexes which facilitate the Ce^3+^ to Ce^4+^ oxidation process,^74^ especially at the surface–solution interface of the REE minerals.^75^ As Ce^4+^ is incompatible within the carbonate structure, it combines with oxygen, forming the cerianite (CeO_2_) oxide.

Our experimental data shows that the Fe^2+^ to Fe^3+^ oxidation initiated prior to the Ce^3+^ to Ce^4+^ oxidation process, revealing a lower energy barrier required for the former compared to the later under our experimental conditions. However, both processes happened simultaneously during most of the duration of the experiment. As both oxidation processes progressed, the pH lowered, dissolving the host and the REE carbonates. At the end of the reaction, the only REE remaining in solid-state was Ce in the cerianite, as well as a small amount of Dy present as impurities in the structure of cerianite. Our experimental design (sealed hydrothermal reactors containing a limited atmosphere, under conditions of autogenous pressure) created an oxidizing environment that favoured the oxidation of Ce^3+^ to Ce^4+^ reaction. Had our experiments been conducted using anoxic water and a pure N_2_ atmosphere, the presence of carbonates such as siderite would have also buffered the pH of the aqueous solution, increasing the stability of the HCO_3_^−^ ions relative to CO_2_. However, these changes would not have a significant influence on the redox state and the formation of Fe and Ce oxides (goethite, hematite and cerianite) would most likely not have occurred. Consequently, Ce would have been incorporated into the newly formed kozoite and bastnasite as Ce^3+^ along with the other REEs in the system. However, in the studied conditions, Ce had a significant impact of the kinetics of the replacement reactions, showing that the rate of decarbonation is dependent on the ratio of Ce relative to other REE in solution. In the PAAS solution, where Ce is dominant compared to the other REE, the higher concentration of Ce leads to a faster release of CO_2_, resulting in quicker decarbonation and dissolution of all the carbonates. Consequently, the full decarbonation process can occur in less than 24 hours. In contrast, in the equal concentration experiments (where REE are present in equal concentrations) all carbonates dissolved after 24 hours.

The replacement of kozoite and bastnasite is also enhanced by the smaller molar volume of cerianite (23.86 cm^3^ mol^−1^) compared to kozoite (45.59 cm^3^ mol^−1^) or bastnasite (44.91 cm^3^ mol^−1^) which increased the porosity of the parent phase.^71^ This porosity helps maintain supersaturation levels at the carbonate–solution interface, allowing the replacement reaction to continue and releasing REE back into the solution. Some REE, such as Dy^3+^ and Nd^3+^, have ionic radii similar to Ce^4+^ and can be incorporated into cerianite as impurities. Depending on the Ce concentration in the fluid, the crystallization of cerianite could trigger the formation of symplectic textures, as described by Maddin *et al.* (2024).^37^ In these textures, cerianite grows into the nanometer-sized pores created by the dissolution of the REE carbonate.^76^Fig. 9 and 14 may indicate the early development of these symplectic textures at the nanoscale.

In natural systems, REE-bearing-carbonatites form through the immiscibility of carbonate–silicate magma and through the fractional crystallization of carbonate minerals from carbonatite magma. The REE form complexes and the magmatic hydrothermal system enables their migration. REE circulation is further promoted by fractures in the deposit, which also provide the space necessary for REE-mineral precipitation. Our hydrothermal replacement experiments in REE-aqueous solutions offers insight into the potential mechanisms and conditions needed for the possible formation of REE-minerals. The differences in molar volumes observed in our experiments, along with the increasing porosity they created, can be correlated to the fractures found in natural ore deposits. As many of the world's carbonatites also host hematite-bearing ores, the use of siderite as our reactant was ideal. The REE uptake by the iron oxides formed was negligible in our experiments; however, further investigation into the use of oxidation processes to separate Fe from REE-bearing ores could have significant potential for improving ore processing techniques and enhancing the economic viability of mining operations.

## Conclusion

The interaction between siderite and multi-component rare earth element (La, Ce, Pr, Nd and Dy) – bearing aqueous solutions resulted in the formation of iron oxides, goethite (α-Fe^3+^O(OH)) and hematite (Fe_2_O_3_), metastable REE-bearing minerals kozoite (REE(CO_3_)(OH)) and bastnasite (REE(CO_3_)(OH,F)), and the rare earth oxide cerianite (CeO_2_). The newly formed phases were the consequence of two oxidation processes, Fe^2+^ to Fe^3+^, promoting goethite and hematite crystallisation, and Ce^3+^ to Ce^4+^, resulting in cerianite formation. Both processes were found to be highly dependent on temperature. At low temperatures, the formation of REE carbonates was inhibited due to a coating of goethite layer that isolated the siderite and created a situation of partial equilibrium. At higher temperatures, the dissolution of siderite was promoted and differences in solubility allowed for the formation of kozoite and bastnasite. However, the continuing oxidation processes acidified the solution, initiating the dissolution of all carbonates and resulted in hematite and cerianite being the final phases in our system.

## Data availability

The data that support the findings of this study are available from the corresponding author upon reasonable request.

## Conflicts of interest

The authors declare no conflict of interest.

## Supplementary Material

RA-014-D4RA05212A-s001
