# Supplementary material for: Transient crystallisation of rare earth carbonates during the hydrothermal oxidation of siderite
Source: RSC Adv. 2024 Nov 5;14(48):35305–22. doi: 10.1039/d4ra05212a (PMC11536270; doi:10.1039/d4ra05212a)

Supplementary Information (SI) for RSC Advances.
This journal is © The Royal Society of Chemistry 2024

Supporting information for the manuscript:

Transient crystallisation of rare earth carbonates during the hydrothermal oxidation of siderite

Maddin, M.*[1], Rateau, R.[1], Szucs, A.M.[1], Terribili, L.[1], and Rodriguez-Blanco, J.D.[2]

[1] Department of Geology, School of Natural Sciences, Trinity College Dublin, Dublin 2, Ireland
[2]iCRAG, Department of Geology, School of natural Sciences, Trinity College Dublin, Dublin 2, Ireland

Figure SI-1. A preferential uptake of Nd and La with Ce in the PAAS solution was observed at 165 ° C after 24 hours.

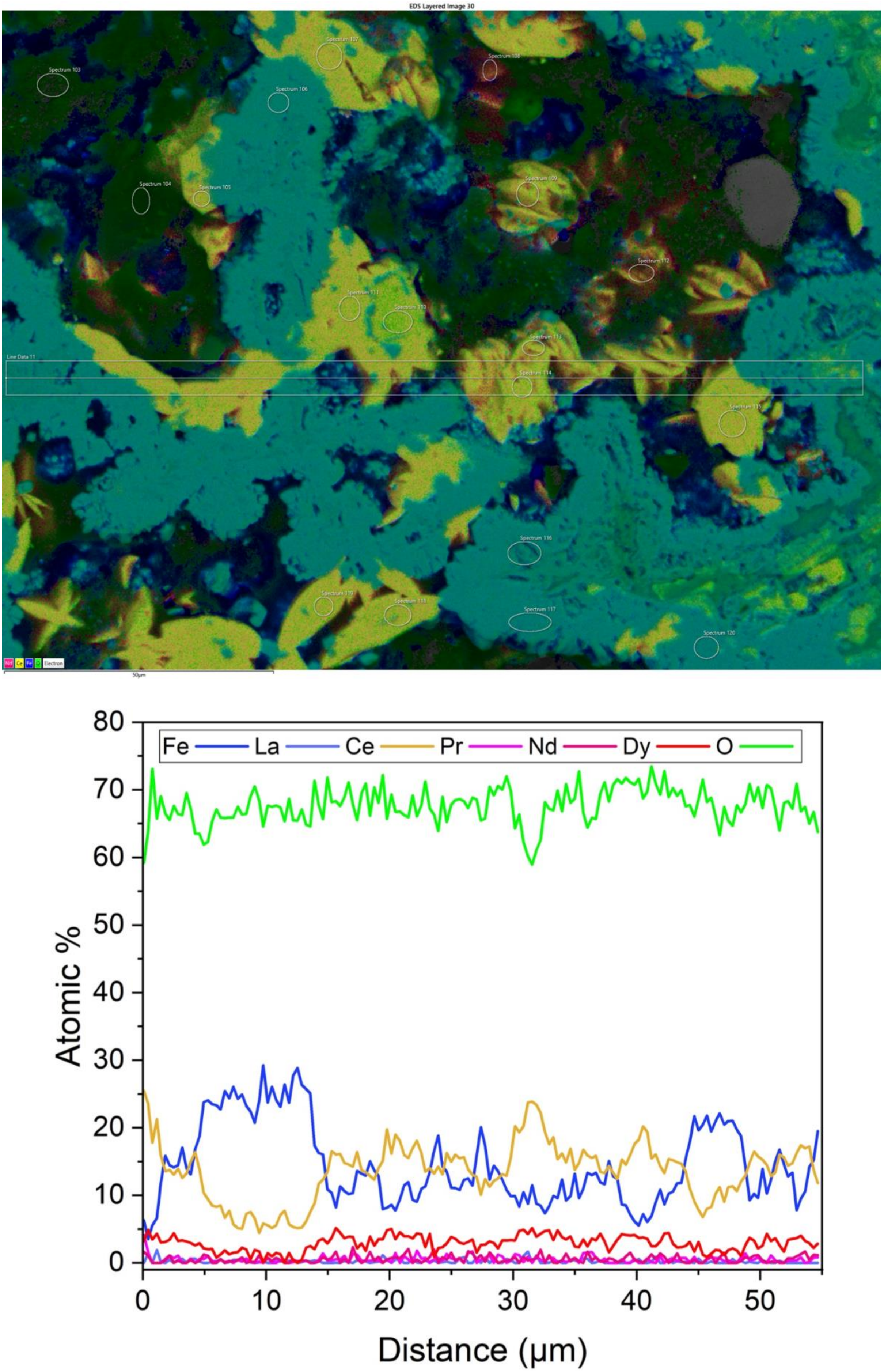

PAAS 165 °C 24 hrs	C	O	Fe	La	Ce	Pr	Nd	Dy	Total
Spectrum 103	0.00	75.27	23.87	0.08	0.76	0.00	0.00	0.20	100.00
Spectrum 104	29.68	59.36	9.15	0.29	0.89	0.19	0.40	0.03	100.00
Spectrum 105	28.86	57.73	1.64	2.14	5.86	0.69	2.78	0.30	100.00
Spectrum 106	25.13	50.25	23.85	0.14	0.37	0.05	0.19	0.01	100.00
Spectrum 107	28.93	57.86	0.95	2.34	6.14	0.69	2.77	0.32	100.00
Spectrum 108	17.07	34.14	12.82	6.22	17.89	2.23	8.72	0.90	100.00
Spectrum 109	28.35	56.70	1.37	2.56	6.76	0.80	3.11	0.35	100.00
Spectrum 110	29.09	58.18	1.03	3.60	5.43	0.48	1.94	0.25	100.00
Spectrum 111	28.98	57.96	0.99	2.68	6.02	0.56	2.50	0.32	100.00
Spectrum 112	22.43	44.85	6.66	4.85	13.09	1.48	6.04	0.61	100.00
Spectrum 113	28.47	56.93	1.68	2.40	6.43	0.77	3.01	0.31	100.00
Spectrum 114	29.01	58.03	0.64	2.67	6.11	0.67	2.54	0.32	100.00
Spectrum 115	28.93	57.86	0.73	2.59	6.23	0.64	2.69	0.33	100.00
Spectrum 116	24.66	49.32	25.46	0.13	0.29	0.04	0.08	0.03	100.00
Spectrum 117	24.82	49.64	25.18	0.07	0.18	0.02	0.07	0.02	100.00
Spectrum 118	28.83	57.66	2.02	2.47	5.63	0.59	2.47	0.33	100.00
Spectrum 119	29.07	58.14	0.61	2.62	6.03	0.61	2.61	0.32	100.00
Spectrum 120	24.83	49.67	24.87	0.11	0.30	0.05	0.13	0.04	100.00

Figure SI-2. Dy incorporation into cerianite in the PAAS solution at 205 °C after 1 week and in the non-PAAS solution at 205 °C after 24 hours

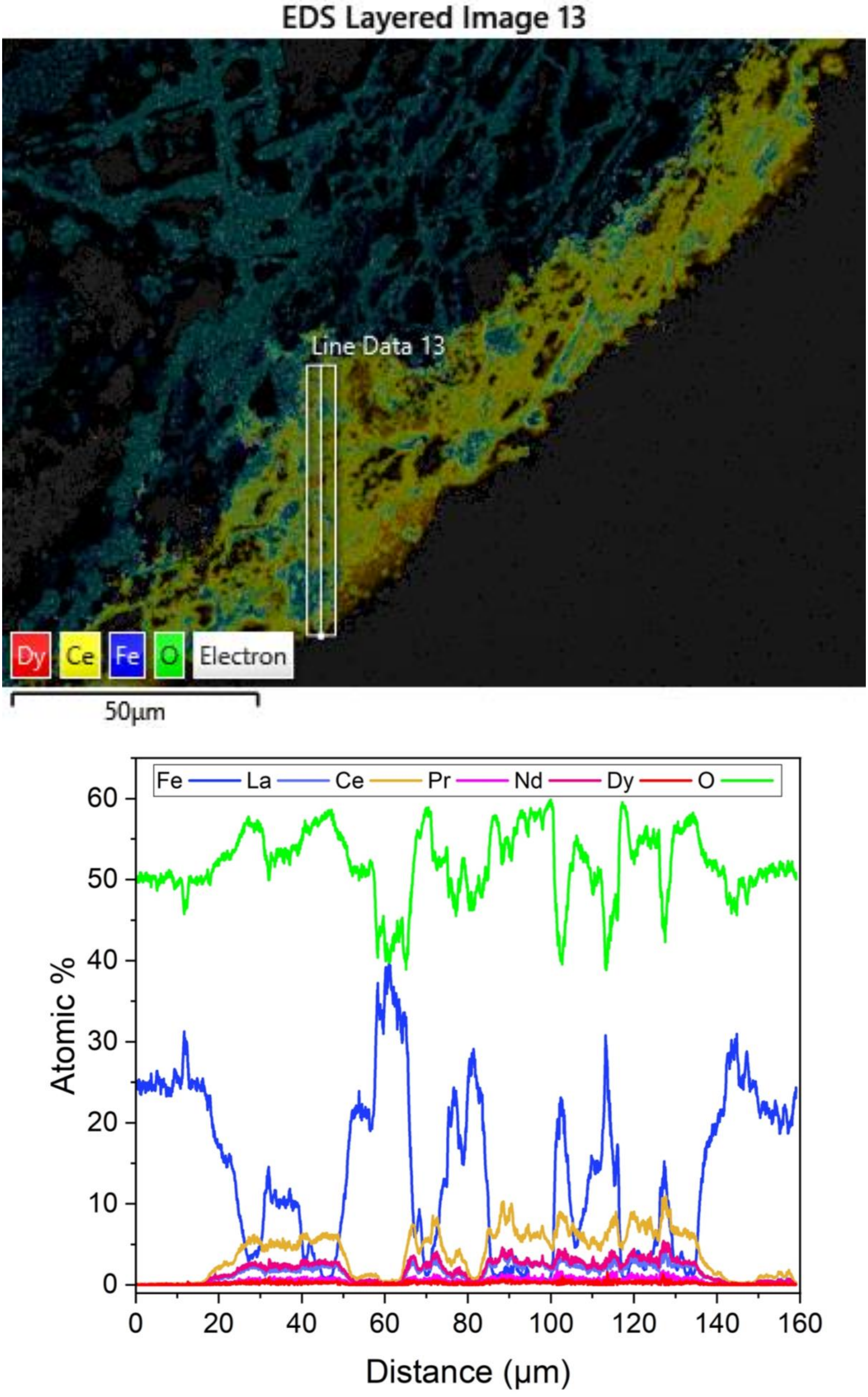

Figure SI-3. Dy incorporation into cerianite in the equal concentration solution at 205 °C after 24 hours, REE concentration in the iron oxides was below the SEM-EDS detection limit, at <0.1%

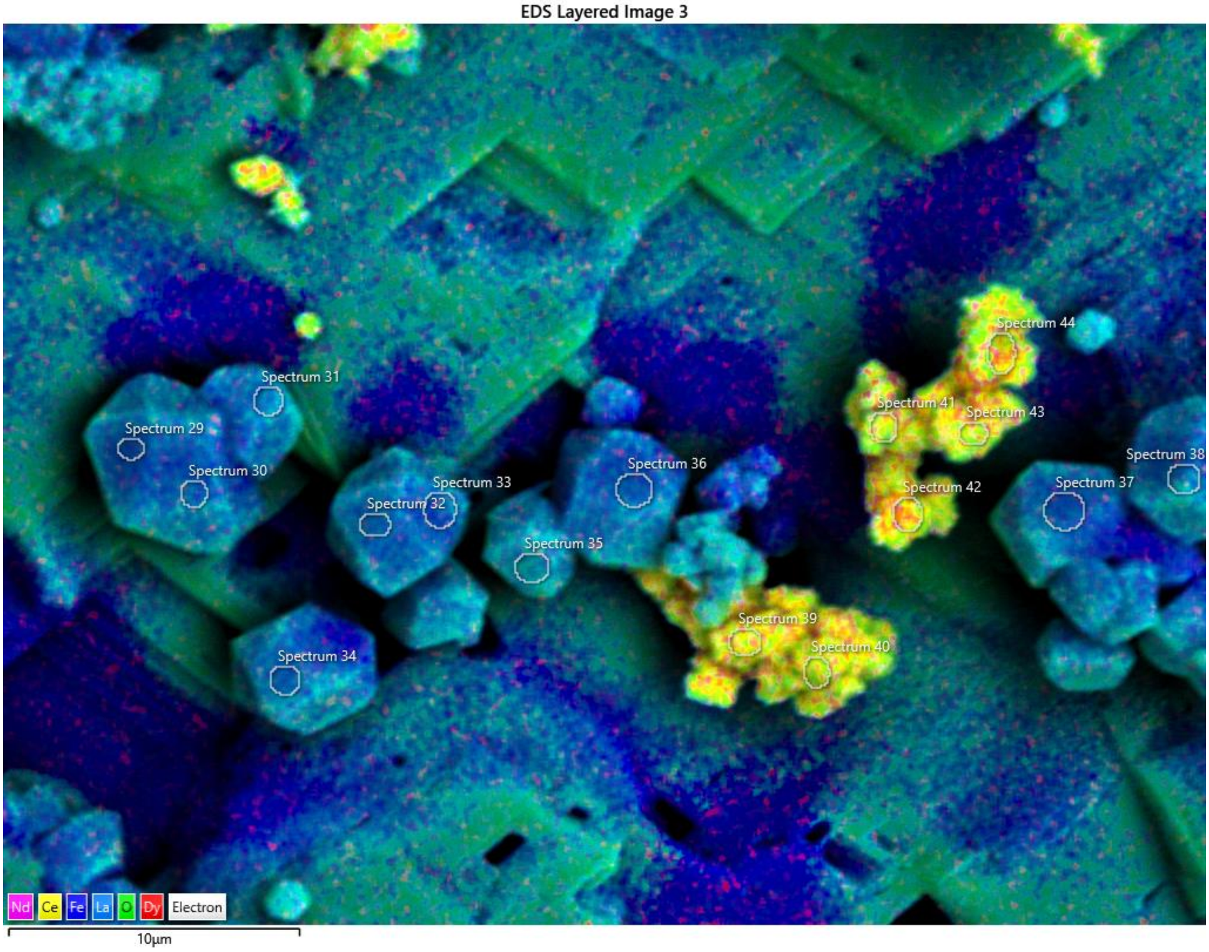

Equal Conc. 205 °C 24 hrs	C	O	Fe	La	Ce	Pr	Nd	Dy	Total
Spectrum 29	18.48	36.96	44.64	0.00	0.06	0.05	0.00	0.00	100.00
Spectrum 30	19.53	39.06	41.21	0.10	0.05	0.08	0.04	0.00	100.00
Spectrum 31	20.30	40.60	38.92	0.01	0.11	0.02	0.00	0.04	100.00
Spectrum 32	19.77	39.54	40.37	0.01	0.14	0.04	0.00	0.15	100.00
Spectrum 33	18.74	37.47	43.31	0.03	0.08	0.00	0.02	0.39	100.00
Spectrum 34	19.25	38.49	42.21	0.06	0.14	0.02	0.04	0.00	100.00
Spectrum 35	22.89	45.79	30.91	0.00	0.22	0.12	0.02	0.09	100.00
Spectrum 36	18.84	37.68	43.26	0.05	0.33	0.07	0.00	0.00	100.00
Spectrum 37	18.59	37.17	43.90	0.00	0.40	0.09	0.03	0.00	100.00
Spectrum 38	20.87	41.73	36.86	0.06	0.31	0.09	0.00	0.13	100.00
Spectrum 39	24.39	48.78	11.84	0.00	11.49	0.57	0.64	2.48	100.00
Spectrum 40	24.29	48.58	14.21	0.03	10.11	0.13	0.21	2.44	100.00
Spectrum 41	23.68	47.36	15.76	0.13	9.83	0.64	0.55	2.06	100.00
Spectrum 42	21.08	42.17	16.23	0.40	16.14	0.04	0.38	3.56	100.00
Spectrum 43	22.61	45.21	17.30	0.29	11.33	0.22	0.35	2.70	100.00
Spectrum 44	21.36	42.72	18.61	0.00	14.05	0.28	0.25	2.95	100.00

Figure SI-4. In the equal concentration experiments (A), the uptake of REEs by the crystallising carbonate occurs in the same ratio as their composition in the aqueous solution, indicating that the crystallising carbonate mirrors the REE composition of the surrounding solution. Conversely, in the PAAS solution experiments (B), the REE ratio is different compared to the equal concentration, but still reflects the higher concentration of La, Ce and Nd in the original solution.

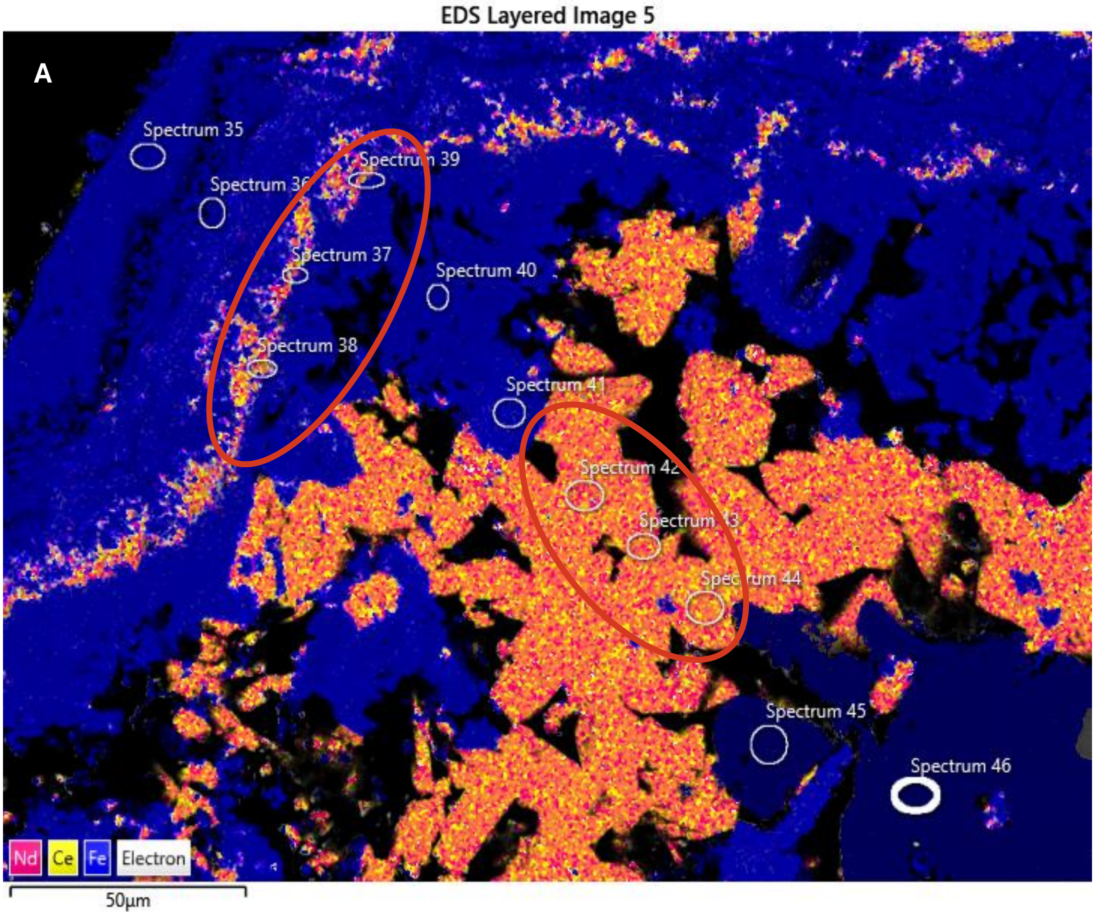

A. Equal Conc. 165 °C 24 hrs	O	Fe	La	Ce	Pr	Nd	Dy	Total
Spectrum 35	60.68	39.11	0.00	0.12	0.08	0.10	0.00	100.00
Spectrum 36	61.95	36.94	0.19	0.21	0.21	0.15	0.34	100.00
Spectrum 37	69.73	22.60	1.37	1.02	1.21	1.57	2.50	100.00
Spectrum 38	70.56	18.75	1.91	2.91	2.47	2.22	1.18	100.00
Spectrum 39	66.27	27.92	0.82	1.04	1.17	1.27	1.50	100.00
Spectrum 40	60.89	39.07	0.14	0.14	0.00	0.05	0.00	100.00
Spectrum 41	64.08	33.32	0.28	0.53	0.46	0.38	0.95	100.00
Spectrum 42	77.41	3.16	4.22	3.52	3.95	4.42	3.33	100.00
Spectrum 43	78.47	1.00	4.04	3.78	5.16	4.80	2.75	100.00
Spectrum 44	78.89	0.65	4.38	4.13	5.04	4.42	2.49	100.00
Spectrum 45	71.56	28.27	0.03	0.10	0.01	0.00	0.12	100.00
Spectrum 46	71.25	28.59	0.04	0.14	0.03	0.00	0.04	100.00

EDS Layered Image 30

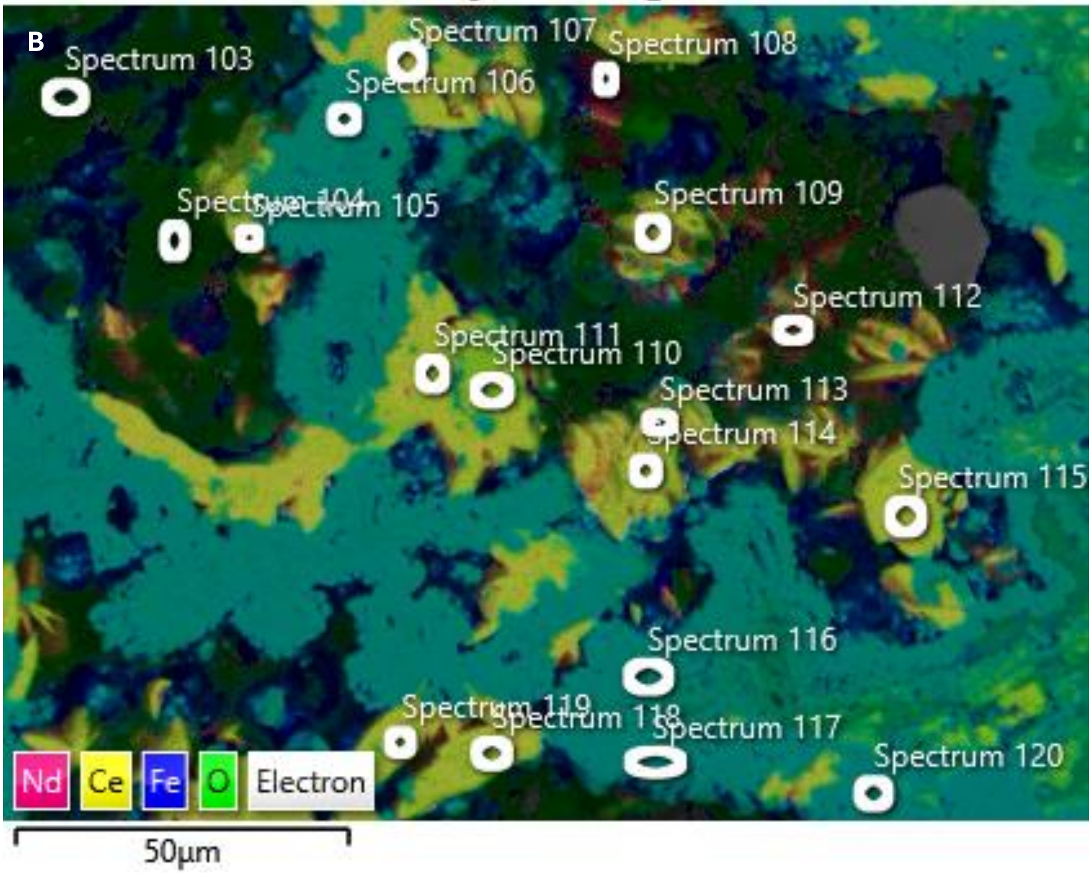

B. PAAS 165 °C 24 hrs	C	O	Fe	La	Ce	Pr	Nd	Dy	Total
Spectrum 103	0.00	75.27	23.87	0.08	0.76	0.00	0.00	0.20	100.00
Spectrum 104	29.68	59.36	9.15	0.29	0.89	0.19	0.40	0.03	100.00
Spectrum 105	28.86	57.73	1.64	2.14	5.86	0.69	2.78	0.30	100.00
Spectrum 106	25.13	50.25	23.85	0.14	0.37	0.05	0.19	0.01	100.00
Spectrum 107	28.93	57.86	0.95	2.34	6.14	0.69	2.77	0.32	100.00
Spectrum 108	17.07	34.14	12.82	6.22	17.89	2.23	8.72	0.90	100.00
Spectrum 109	28.35	56.70	1.37	2.56	6.76	0.80	3.11	0.35	100.00
Spectrum 110	29.09	58.18	1.03	3.60	5.43	0.48	1.94	0.25	100.00
Spectrum 111	28.98	57.96	0.99	2.68	6.02	0.56	2.50	0.32	100.00
Spectrum 112	22.43	44.85	6.66	4.85	13.09	1.48	6.04	0.61	100.00
Spectrum 113	28.47	56.93	1.68	2.40	6.43	0.77	3.01	0.31	100.00
Spectrum 114	29.01	58.03	0.64	2.67	6.11	0.67	2.54	0.32	100.00
Spectrum 115	28.93	57.86	0.73	2.59	6.23	0.64	2.69	0.33	100.00
Spectrum 116	24.66	49.32	25.46	0.13	0.29	0.04	0.08	0.03	100.00
Spectrum 117	24.82	49.64	25.18	0.07	0.18	0.02	0.07	0.02	100.00
Spectrum 118	28.83	57.66	2.02	2.47	5.63	0.59	2.47	0.33	100.00
Spectrum 119	29.07	58.14	0.61	2.62	6.03	0.61	2.61	0.32	100.00
Spectrum 120	24.83	49.67	24.87	0.11	0.30	0.05	0.13	0.04	100.00

Figure SI-5. Powder XRD patterns of goethite (G) in the equal concentration (non-PAAS) and PAAS experiments at 50 °C after 24 hours and 8 weeks (*Bragg peaks that have not been assigned to any phase correspond to siderite). The diagram in the bottom area shows the standard XRD patterns of the phases found in these experiments.

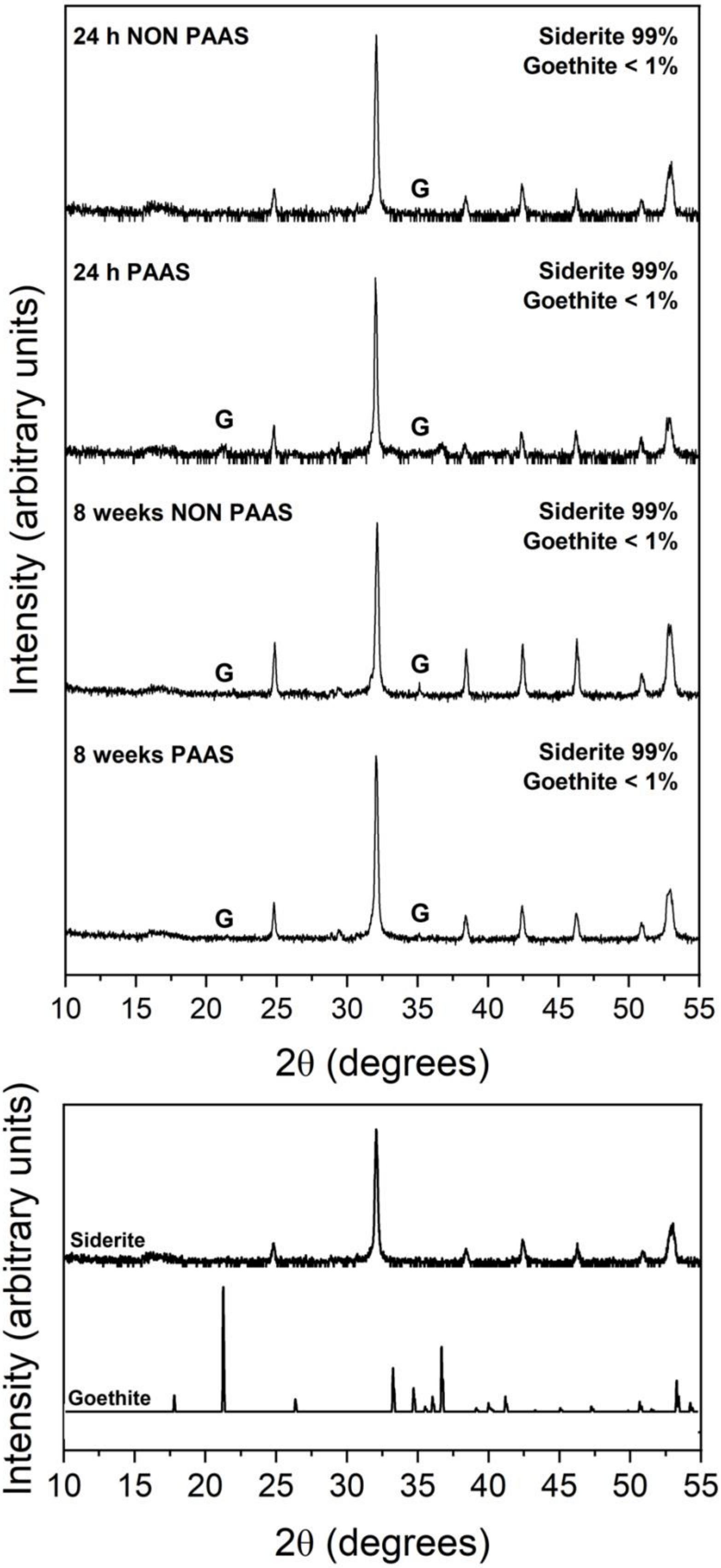

Figure SI-6. Powder XRD patterns of goethite (G), hematite (H), kozoite (K), hydroxylbastnasite (B) and cerianite (C) in the equal concentration (non-PAAS) and PAAS experiments at 165 °C after 24 hours and 2 weeks (*Bragg peaks that have not been assigned to any phase correspond to siderite) The diagram in the right area shows the standard XRD patterns of the phases found in these experiments.

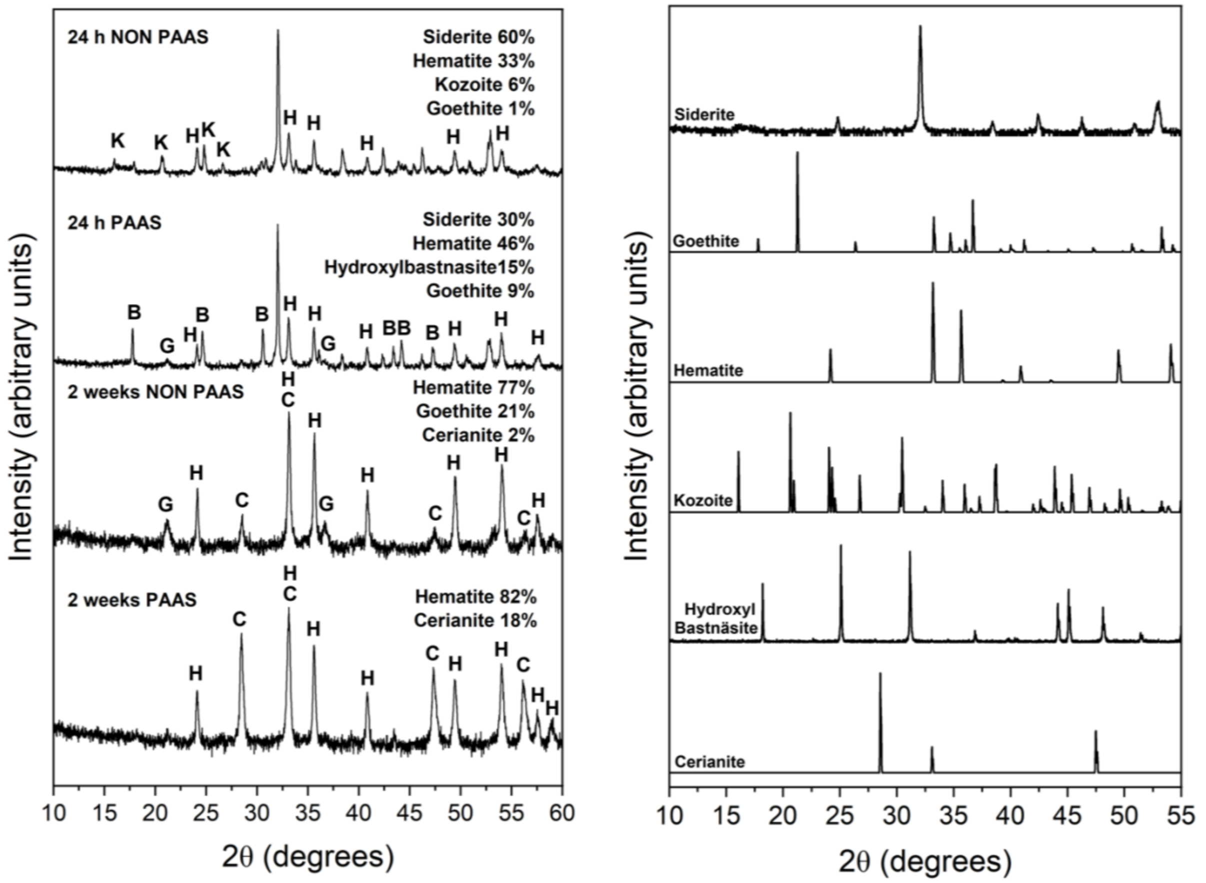

Figure SI-7. Powder XRD patterns of, hematite (H), hydroxylbastnasite (B) and cerianite (C) in the equal concentration (non-PAAS) and PAAS experiments at 205 °C after 24 hours (*Bragg peaks that have not been assigned to any phase correspond to siderite). The diagram in the bottom area shows the standard XRD patterns of the phases found in these experiments.

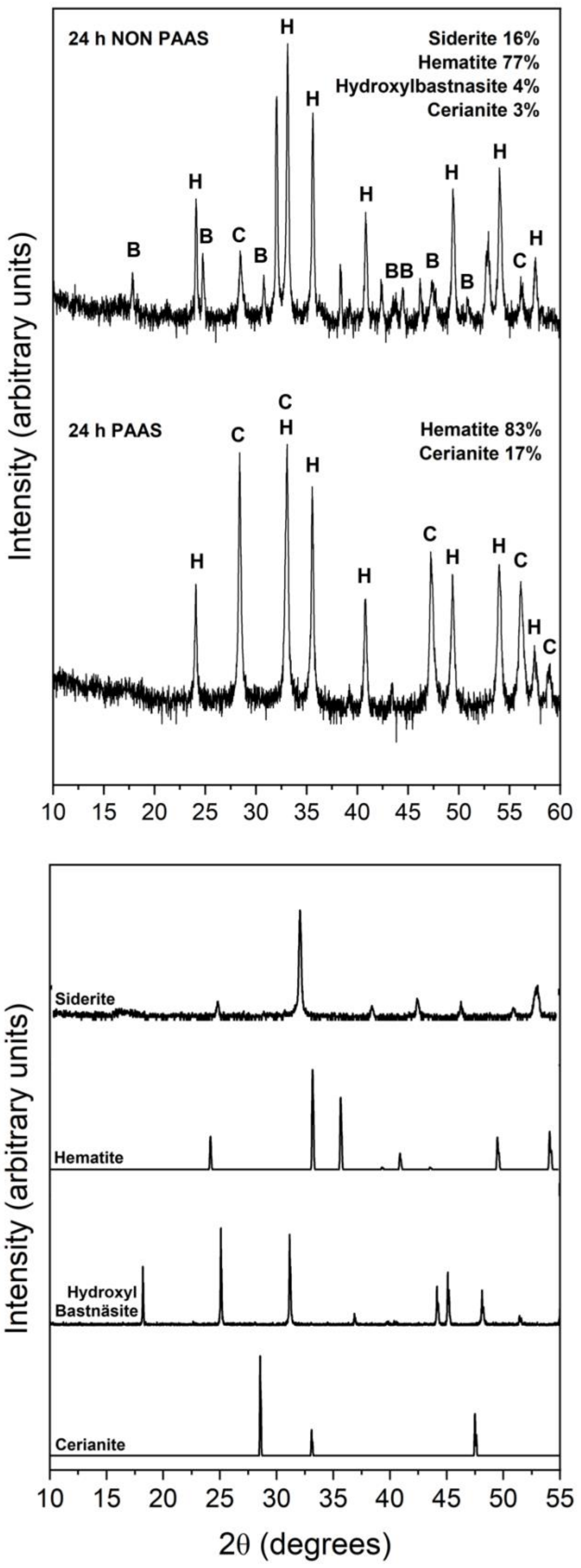

Supplement: RA-014-D4RA05212A-s001 [file RA-014-D4RA05212A-s001.pdf]
